# Safety, cost and environmental impact of reprocessing high risk single-use medical devices: a systematic review and meta-analysis 

**DOI:** 10.3205/dgkh000554

**Published:** 2025-06-06

**Authors:** Niamh McGrath, Catherine Waldron, Ailish Farragher, Cathal Walsh, Julie Polisena

**Affiliations:** 1 Evidence Centre, Health Information and Evidence Directorate, Health Research Board, Dublin, Ireland; 2 Biostatistics Unit, School of Medicine, Trinity College Dublin, Dublin, Ireland; 3 Health Technology Assessment Division, International Federation for Medical and Biological Engineering, Ottawa, Ontario, Canada

**Keywords:** systematic review, cardiac catheterization, defibrillators, implantable, pacemaker, artificial, patient safety, disinfection

## Abstract

**Aim:**

To estimate the safety, financial and environmental effects of reprocessing high risk SUMDs.

**Methods:**

Systematic review (PROSPERO ID: CRD42022365642) of primary trial and observational studies of human participants receiving reprocessed high risk SUMDs compared with first use of identical SUMDs. Reprocessing was defined as cleaning, disinfection, and sterilisation or related procedures, and function and safety testing. Items were sourced via database, grey literature and supplemental searching of English and German language sources. Included studies were quality appraised and primary outcomes (direct patient safety; indirect financial costs; environmental impacts) GRADE (Grade of Recommendation, Assessment, Development and Evaluation) assessed. Narrative synthesis and where feasible, meta-analysis were undertaken.

**Results:**

Ten studies (N=2,657 participants) examined two implantable (pacemaker, defibrillator) and three catheterisation (electrophysiology polyurethane, ablation and balloon) devices. Safety outcomes were available for both device types and cost outcomes were available for catheterisation devices. Except for one older study, there were no statistically significant differences in the odds of examined safety outcomes between new and once reprocessed SUMDs. Meta-analysis of catheterisation devices resulted in similar results (Infections: OR=0.67, 95% CI: 0.37–1.20,* p*=0.18; Battery depletion: OR=0.2.29, 95% CI: 0.83–6.31, *p*=0.11). One study of balloon catheterisation devices which accounted for indirect costs reported savings of CAN$ 129 per patient. The certainty of evidence, using the GRADE assessment, for each outcome was very low.

**Conclusion:**

We found no evidence of additional adverse safety outcomes for once reprocessed cardiac catheterisation or implantable cardiac SUMDs. However, our confidence that the same findings would be observed in future studies is very low. There was insufficient evidence to establish the cost-effectiveness or environmental impacts of reusing cardiac catheterisation or implantable SUMDs. High-quality randomised controlled trials, analyses of national device reprocessing surveillance systems, cost-effectiveness studies, and life cycle assessments are required in order to facilitate better comparison across devices and reprocessing contexts.

## Zusammenfassung

**Zielsetzung:**

Abschätzung der sicherheitstechnischen, finanziellen und ökologischen Auswirkungen der Aufbereitung von Hochrisiko Single-Use-Medizinprodukten (single-use- medical-devices, SUMDS).

**Methode:**

Systematische Überprüfung (PROSPERO ID: CRD42022365642) von Primärstudien und Beobachtungsstudien über Patienten, die aufbereitete Hochrisiko-SUMDs, im Vergleich zur Erstverwendung identischer SUMDs erhielten. Die Aufbereitung wurde definiert als Reinigung, Desinfektion und Sterilisation oder verwandte Verfahren sowie Funktions- und Sicherheitstests. Die Artikel wurden über Datenbanken, graue Literatur und über eine ergänzende Suche in englisch- und deutschsprachigen Quellen beschafft. Die eingeschlossenen Studien wurden einer Qualitätsprüfung unterzogen und die primären Ergebnisse (direkte Patientensicherheit, indirekte finanzielle Kosten, Umweltauswirkungen) nach GRADE (Grade of Recommendation, Assessment, Development and Evaluation) bewertet. Es wurden eine narrative Synthese und, soweit möglich, eine Meta-Analyse durchgeführt.

**Ergebnisse:**

In zehn Studien (n=2657 Teilnehmer) wurden zwei implantierbare Devives (Herzschrittmacher, Defibrillator) und drei Katheterer (Elektrophysiologie-Polyurethan, Ablation und Ballon) untersucht. Für beide Gerätetypen lagen Sicherheitsprüfergebnisse und für die Katheter lagen Kostenergebnisse vor. Mit Ausnahme einer älteren Studie gab es keine signifikanten Unterschiede in der Wahrscheinlichkeit untersuchter Sicherheitsergebnisse zwischen neuen und einmal aufbereiteten SUMDs. Die Meta-Analyse von Kathetern ergab ähnliche Ergebnisse (Infektionen: OR=0,67, 95%CI: 0,37–1,20, p=0,18; Batterieerschöpfung: OR=0,2,29, 95% CI: 0,83–6,31, p=0,11). Eine Studie über Ballonkatheter, in der die indirekten Kosten berücksichtigt wurden, ergab Einsparungen von 129 CAN$ pro Patient. Die Evidenz, die sich aus der GRADE-Bewertung ergibt, war für jedes Ergebnis sehr gering.

## Introduction

During the 1980s, the number of medical devices produced, labelled, and marketed by manufacturers “for single use only” increased [1], [2]. Demand for these single-use medical devices (SUMDs) was driven by the development of SUMDs with smaller lumens and more intricate, delicate working mechanisms and efforts to reduce the risks of cross-contamination from one patient to the next [1]. Manufacturers may market devices as single-use where:

- There are concerns about the feasibility of making the device with reusable materials and achieve the desired function,
- it is impossible to design a device which achieves the desired function while allowing patient-safe reprocessing, or
- manufacturers wish to control or limit their liability for device failure [1].

In order to reduce hospital costs, SUMD reuse is practiced globally, including in Europe [3]. Prior to reuse, devices are reprocessed, which is defined in European legislation as “a process carried out on a used device in order to allow its safe reuse, including cleaning, disinfection, sterilisation and related procedures, as well as testing and restoring the technical and functional safety of the used device” [4], with a similar definition employed in medical device research [5]. In developed countries, proponents of SUMD reprocessing argue that the practice has economic and environmental benefits [3] and that regulating SUMD reprocessing reduces the risk of unsafe interventions and resultant infections reported in developing and transitional countries [3]. 

Currently, the greatest volume of research evidence is available for implantable cardiac devices [6], [7], [8]. A 2011 systematic review reported increased odds of device failure but not of infections among patients receiving reprocessed versus new devices [6] and more recent reviews published in 2014 and 2021 respectively reported no differences in patient deaths (none reported), infection rates or device related failures [7], [8]. A 2004 health technology assessment of the safety and effectiveness of the reuse of SUMDs found that the evidence for high risk SUMDs, specifically cardiac catheterisation, and cardiac cannula devices, was limited and inconclusive. Specifically, percutaneous transluminal coronary angioplasty (PTCA) catheters were considered safe to reprocess but results became mixed when in-vitro studies were also considered [9]. Twenty years later, a 2024 rapid review concluded that the evidence base for high risk, or critical, SUMD reprocessing in surgical settings was insufficient to determine whether the practice affects patient safety [10]. As with implantable cardiac device syntheses [6], [7], [8] reprocessing quality assurance standards e.g. regulatory oversight, was not recorded or its impact evaluated [10]. Regarding cost-effectiveness, although the European medical device industry have stated that cost savings may reach 50% for certain devices (e.g. electrophysiology or ablation catheters), and up to 90% when reprocessing is done in-house [1], the available research evidence from two systematic reviews on the topic have concluded that the cost-effectiveness of SUMD reprocessing is inconclusive due to a paucity of high-quality, appropriately designed studies [2], [11]. Life cycle assessment studies examine the environmental impact of a medical device from its development to disposal. To our knowledge, such studies examining the environmental impact of high risk SUMD reprocessing have not yet been synthesised.

### Objectives

In 2023, the Health Research Board completed an evidence review requested by the Department of Health in Ireland on the safety, financial costs and environmental impacts of reprocessing SUMDs. The current article presents the findings of the evidence review concerning risk class III devices. It also incorporates the results of a more updated search strategy than that used in the original review. Risk class III devices convey the greatest potential risk to patient safety [12] and are among the most expensive medical devices to produce [13]. Therefore, a synthesis of the safety and cost-effectiveness of these devices is of particular interest. 

The aims of the review are to:

1. Identify the SUMDs safe to reprocess in line with the 2017 EU medical device regulation and other related approaches, and
2. Synthesise the safety, financial and environmental consequences of high risk class SUMD reprocessing in line with the 2017 EU medical device regulation and other related approaches and any differences across SUMD type.

## Methods

### Review design 

A systematic review was conducted [14] and reported according to the Preferred Reporting Items for Systematic Reviews and Meta-Analyses (PRISMA) criteria [15], [16]. Procedures were consistent with guidance on systematic reviews with cost and cost-effectiveness outcomes [17]. The original study protocol was registered on the International Prospective Register of Systematic Reviews (PROSPERO) (ID: CRD42022365642). The full review included results of both laboratory and clinical studies, and all SUMD risk classes. In this article, we present the results of clinical studies of high risk SUMDs only.

### Literature search strategy 

We identified peer-reviewed published literature by searching the following bibliographic databases: EMBASE, MEDLINE (Ovid platform), Dimensions, and the Cochrane Library (John Wiley and Sons Inc.). The search strategy consisted of controlled vocabulary, specifically the National Library of Medicine’s MeSH (medical subject headings), and keywords. The peer reviewed search centred on five concepts: single-use medical devices, reprocessing, environmental impacts, safety and/or adverse outcomes, and cost and cost-effectiveness. Supplementary and grey literature searches were also performed. We limited the search to English and German language documents, owing to Germany’s significant experience in SUMD reprocessing. Searches were undertaken between 25 July and 23 September 2022 and updated in January 2024. The search strategy is available in Attachment 1.

### Eligibility criteria 

The eligibility criteria were defined using the Population Intervention Comparison Outcomes Study design (PICOS) framework (Attachment 2). SUMDs included devices and purpose-built components thereof exposed to human cells, bacteria and/or viruses. In order to comply with the EU MDR and to ensure health system comparability, primary studies of any healthcare facility using reprocessed SUMDs in Organisation for Economic Co-operation and Development (OECD) or EU member states only were eligible. Reprocessing was defined using the definition provided in EU legislation ([4], p.18). Studies must have included at least one type of primary outcome of interest (i.e., direct patient safety, indirect financial costs and/or environmental impacts) and compared outcomes with first use of the same SUMD. We did not include systematic review studies because the search terms employed in existing systematic reviews included terms inconsistent with our definition of reprocessing or were not reported, so we could not be certain that the evidence included in systematic reviews would reflect reprocessing as defined in this systematic review [2], [11]. Conference abstracts, letters and editorials and animal studies were excluded. 

### Article selection 

Following deduplication in EndNote, two of three possible screeners (NMG, LK, CW) screened each item. Where it was unclear about individual study eligibility due to missing information at full text screening stage, study authors were contacted to seek clarification. If study authors did not respond within two weeks after the initial email and one week after a reminder email, the study was excluded. No studies reporting environmental outcomes were identified and therefore extraction and quality appraisal related to these outcomes are not described further. 

### Data extraction and outcome selection 

Study data were extracted independently by two of four reviewers (NMG, CW, LK, ÁT) into bespoke extraction forms, tailored to the study design and subsequently agreed by the two reviewers. Third-party arbitration was used to resolve disagreements. 

Primary outcomes were those which directly impacted patient safety, such as complications and accounted for indirect reprocessing costs. Safety and cost outcomes were selected for extraction by the review team based on their prevalence across device-specific studies, objective measurement, and directness to patient safety and transparency of reporting, costs used and cost sources (Attachment 3). Major complications for cardiac catheter device studies were grouped as: evidence of subsequent myocardial infarction (i.e., acute and subacute), evidence of requirement for emergent percutaneous or surgical revascularisation of the target vessel, death, or thrombus. Minor complications for cardiac catheter device studies were grouped as pyrogen reactions (i.e., fever, temperature, white blood cell count), creatine kinase, and/or author-labelled minor complications. 

### Quality assessment 

Two reviewers independently assessed the quality of the studies included, with disagreements resolved by consensus. 

Adapted versions of the 27-item Downs and Black [18] and 19-item Consensus Health Economic Criteria list (CHEC-list) [19] were employed to quality appraise randomized and non-randomised studies and economic study designs respectively. The Downs and Black checklist is ranked in the top six quality assessment tools suitable for use in systematic reviews [20] and the CHEC-list was developed by international experts for systematic reviews of full economic evaluations based on effectiveness studies [19]. Details of the adaptations made to the quality appraisal tools are reported in Attachment 4.

### Data analysis and synthesis 

#### Meta-analysis 

For each outcome of interest, we completed an assessment of the feasibility of meta-analysis following published guidance [21], [22] (Attachment 5). Meta-analyses were performed in Review Manager Software version 5.4 using a random-effects model due to study-level variability identified across feasibility assessments [23]. Odds ratios were calculated for categorical outcomes, and mean differences were calculated for continuous outcomes [23]. Heterogeneity was quantified using Higgins and Thompson’s *I^2^* statistic, defined as the percentage of variability in the effect sizes that is not caused by sampling error [23].

#### Narrative synthesis 

A structured reporting of effects was completed, calculating a standardised effect measure for safety outcomes (i.e. odds ratios for categorical outcomes and mean differences for continuous outcomes) including reporting of the number of observed events in the total population (categorical outcomes) and the mean/median with standard deviations (SDs) for continuous events [23]. 

#### Grading of recommendations, assessment, development and evaluations 

The GRADE system was employed to grade the quality of individual review outcomes based on the contributing primary studies. In line with best practice, we applied GRADE assessments to primary review outcomes only [24]. 

## Results

### Search results and included studies 

**Figure 1:**
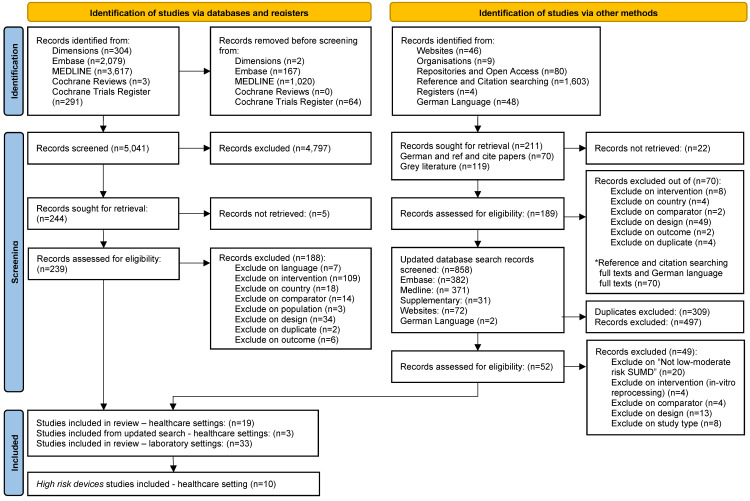
Flow diagram of the study identification and selection process, following Preferred Reporting Items for Systematic Reviews and Meta-Analyses (PRISMA) guidelines

Details of the search results and the PRISMA flow diagram for is reported in Figure 1 (Fig. 1). We identified 10 studies examining three cardiac catheterisation (i.e., electrophysiology polyurethane, ablation and balloon) and two implantable cardiac (i.e., pacemaker, defibrillator) devices. Cardiac catheter devices are used for both diagnostic and therapeutic purposes. Balloon catheters are used to open up blocked arteries and veins during a coronary angioplasty; ablation catheters are used during treatment for atrial fibrillation; and electrophysiology polyurethane catheters are used for recording and pacing the electrical potentials from within the heart [25], [26]. Implantable cardioverter defibrillators are small, battery-powered devices placed in the chest to detect and stop potentially life-threatening abnormal heart rhythms. Pacemakers are small devices that are implanted in the chest to help monitor the heart rate and rhythm and provide pacemaker support when needed [27], [28]. 

### Characteristics of included studies 

The characteristics of included studies are reported in Table 1 (Tab. 1). The studies were undertaken in the EU (n=5), the USA (n=2), Mexico (n=1), Canada (n=1) and the UK (n=1). Safety outcome data were available for both device types and cost outcomes were also available for catheterisation device studies. Apart from one study, all safety studies employed observational designs (n=7) and one cost study employed an economic evaluation design. 

Most catheter devices were reprocessed outside of the hospital (n=3 studies), whereas implantable cardiac devices were reprocessed by hospitals. Two catheterisation device studies followed FDA or EU MDR reprocessing regulations and two other studies (three papers) which were undertaken prior to the introduction of legal regulation followed criteria set by the research teams. In contrast, implantable device studies followed local hospital policies or research team criteria. Three catheterisation device studies compared new devices with those put through multiple (one to six) reprocessing cycles, whereas implantable devices were only reprocessed once. 

### Safety outcomes 

#### Cardiac catheter devices 

As indicated by the meta-analysis feasibility assessment (Attachment 5), no cardiac catheter device safety outcomes were feasible for meta-analysis and were reported narratively (Table 2 (Tab. 2)). One of four catheter studies [29] collecting major complications data reported their higher odds of occurrence in the reused compared with new device group (OR=2.76, 95% CI: 1.41–5.40). There were no significant differences in the odds of occurrence of minor complications in the three studies contributing data for this outcome [30], [31], [29]. No explicit patterns were identified for secondary outcomes (e.g., average procedure time, fluoroscopy time and contrast dye used) with studies favouring single-use, reuse or reporting no differences (Table 2 (Tab. 2)).

Unverdorben et al. [32] reported the safety outcomes by each subsequent reprocessing cycle for up to three reuses. Study authors reported no significant difference in the average procedure time between new devices (9.9, ±6.8) and the first (9.3, ±4.9), second (12.5, ±7.2), and third (11.5, ±1.6) reprocessing cycles (*p*=0.076). Differences reached statistical significance for average fluoroscopy time between new devices (2.6, ±2.8) and the first (2.4, ±1.9), second (3.2, ±2.7), and third (4.2, ±5.4; *p*=0.052) reprocessing cycles. There was no significant difference in the average volume of contrast used between new devices (44, ±32) and the first (40, ±27), second (47, ±26), and third (49, ±29) reprocessing cycles (*p*=0.290). Studies providing safety data were of low to good quality (Table 2 (Tab. 2) and Attachment 4).

#### Implantable cardiac devices 

**Figure 2:**
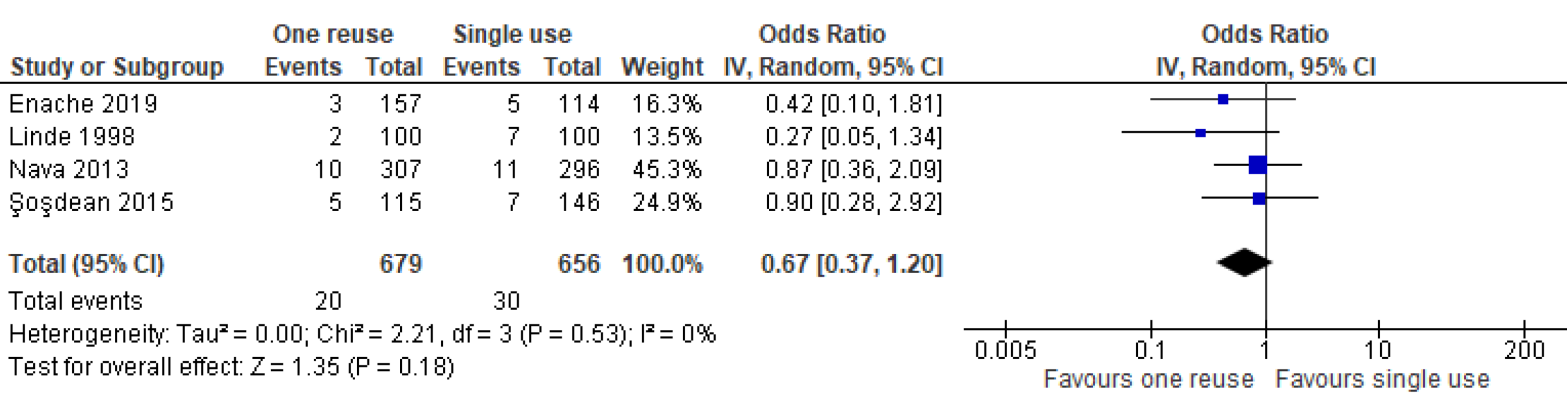
Forest plot of the rate of device-related infections in studies of new devices compared with reused devices

**Figure 3:**
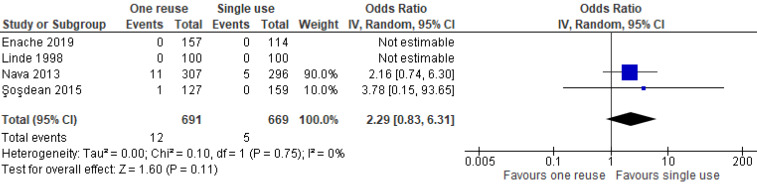
Forest plot of the rate of unexpected battery depletion in studies of new devices compared with reused devices

As indicated by the meta-analysis feasibility assessment (Attachment 5), two of three safety outcomes (infections and unexpected battery depletion) were feasible for meta-analysis. Narrative synthesis for all outcomes provided in Table 2 (Tab. 2) indicate no significant differences in the outcomes assessed between once-reprocessed and new implantable cardiac devices. The results of the meta-analysis were consistent with the narrative summary. Meta-analysis found no statistically significant difference in the occurrence of device-associated infections in the reused device compared with the new device group (20 versus 30 events, *p*=0.180, OR=0.67 [95% CI: 0.37–1.20]; see Figure 2 (Fig. 2). There was no heterogeneity between individual study effect sizes. Meta-analysis found no statistically significant difference in the odds of unexpected battery depletion between the reused device compared with the new device group (12 versus 5 events, p=0.110, OR=2.29 [95% CI: 0.83–6.31]; see Figure 3 (Fig. 3)). There was no heterogeneity between individual study effect sizes.

### Cost outcomes 

Cost outcomes were available for cardiac catheter devices. Two studies provided data derived from direct [33], [29] and indirect costs [33]. Although Mak et al. [33] presented cost models derived from three possible scenarios (“best case”, “likely case”, and “worst case”) based on clinical data reported in Plante et al. [29], we only report cost estimates derived from the “likely case” scenario. Detail of the best- and worst-case scenarios are available in the original study report. Tessarolo et al. [34] estimated costs based on department activity (number of catheters used per year) across Italian hospital cardiology departments compared with single device use. Studies estimated costs in CAN$ [33] and EUR [34] with costs calculated approximately 10 years apart; 5 months during the year 1994 for Mak et al. [33] and 1 year during the year 2004 for Tessarolo et al. [34]. Accounting for indirect costs, savings to departments differed by device [34] and were reported at the individual patient level [33] but the statistical significance of the savings was not estimated (Table 2 (Tab. 2)). Studies providing data on cost differences for cardiac catheter devices were of low and moderate quality (Table 2 (Tab. 2) and Attachment 4). 

### Environmental outcomes 

No studies were identified providing data on the environmental impact of reusing reprocessed cardiac catheter devices or for implantable cardiac devices (Table 1 (Tab. 1)).

### Grading of recommendations, assessment, development and evaluations rating 

Scores and explanatory judgement for each of the three evaluated safety outcomes; major complications for catheterisation devices, infections and unexpected battery depletion for implantable devices and the one cost outcome; total cost difference per patient (catheterisation devices) are displayed in Attachment 6. For all outcomes, the a priori rating was “low”, because most of the evidence for each of the four primary outcomes was derived from observational studies. Each outcome received at least one downgrade. When downgrades were applied, all outcomes received a final rating of very low certainty in the evidence. 

## Discussion

Our systematic review examines SUMD reprocessing across high risk devices and outcome types central to SUMD reprocessing debate [1], [3]. Ten studies examining three catheterisation (i.e., electrophysiology polyurethane, ablation and balloon) and two implantable (i.e., pacemaker, defibrillator) devices were identified. 

The available evidence was insufficient to deem specific SUMDs safe to reprocess following the requirements of the EU MDR or related approaches. The findings were consistent with previous evidence syntheses including cardiac catheter devices which were unable to draw any definitive conclusions about the safety of reprocessing these devices [9], [10], [11], [35].In this review, the one study reporting events for major complications [29] was rated as being of good quality but was not subject to the same level of reprocessing oversight as the other studies collecting data on this outcome did [31], [32]. The occurrence of secondary safety outcomes (minor complications, procedure time, fluoroscopy time, contrast volume used) were conflicting and imprecise, with some studies finding in favour and others against SUMD reprocessing and many studies reporting wide confidence intervals. Based on the results of the meta-analysis, single use implantable devices cannot be safely reprocessed. Although our finding of no additional adverse safety events for once reprocessed versus new implanted cardiac devices was consistent with related systematic reviews on the topic [6], [8] the possibility of unexpected battery depletion appears higher in the reused device group, albeit not significantly so. This is one reason why reprocessing implanted cardiac devices is not advised in Europe [36]. It is also worth noting that none of the clinical studies in this review were designed to be able to capture risk of prion infections [37]. However, we estimate this risk to be low given that most studies in this review involved interventions at sites distant from neurological exposure. 

Regarding research question 2, there were no new costing or cost-effectiveness studies identified since the reviews published by Hailey et al. [11] or Jacobs et al. [2] in 2008. Consequently, our interpretation of the cost-effectiveness research evidence for high risk SUMD reprocessing remains unchanged i.e. direct cost savings of high risk SUMD reprocessing differ across individual devices [2], [11], but the cost-effectiveness of reprocessing high risk SUMDs is unknown [2], [11]. Given that we identified no life cycle assessment studies of high risk devices, the environmental consequences of high risk SUMD reprocessing remain unknown. 

Finally, with respect to similarities and differences across high risk devices, safety was the only outcome examined across both device types. Possibly due to the nature of implantable cardiac devices which remain inside the body, safety outcomes for these devices were followed up for a much longer period, approximately 3 months for cardiac catheters [22], [23], versus 3 years for implantable cardiac devices [26], [27], [28]. 

### Strengths and limitations 

The strengths of this review are its broad focus and the rigorous methods employed. In this article, we present the available published literature on the safety, costs, and environmental impacts of reprocessing the SUMDs which typically convey the greatest risk to patients [4] and are among the costliest to produce [13]. Additionally, for the first time, we attempt to consider the alignment of reprocessing with quality assurance standards in order to help contextualise similarities and differences in the findings between studies of similar high risk SUMDs [6], [8], [9], [10]. By using a modern definition of reprocessing to determine study eligibility for inclusion in this systematic review, we were able to eliminate risks of including studies of similar related practices (e.g. sterilisation only, recycling, reprocessing for single-patient reuse) [6], [8], [9], [10]. For instance, two (of a total of eight) studies included in Kaulback and Horton’s 2024 rapid review of high risk SUMD reprocessing were excluded from our review at screening stage as they did not meet our definition of reprocessing. Specifically, device function testing was not reported by study authors [38] and [39]. By distinguishing between the different “levels” of reprocessing oversight across studies, there was potential to explore trade-offs between reprocessing safety [3] and cost saving outcomes by reprocessing oversight. This was useful because regulating reprocessing often requires outsourcing of reprocessing from hospital Central Sterile Service Departments to reprocessing companies [3]. Conversely, the possibility that excluding studies, which did not define “reprocessing” or report on the reprocessing-related procedures followed could have resulted in missing otherwise eligible items cannot be ruled out. The failure to report this information could add confusion to this topic and authors are encouraged to include these details in their studies. To ensure adequate clinical knowledge of individual SUMDs, advice was sought from the Health Products Regulatory Authority (HPRA), Ireland’s regulatory body for health products, including medical devices. We consulted with a medical cardiology doctor to inform the section of outcomes for cardiac catheter device studies and two UK-based cardiologists active in SUMD reprocessing research peer-reviewed the full review.

Although standardising cost results to a single currency and for the current year to adjust for inflation is common in systematic reviews of economic studies [17], we felt that doing so would not result in comparable costs in this review. This is due to the quality of the cost studies identified, the specific cost outcomes identified (mainly direct costs), and the likely advances in technology and regional differences in costs in the available studies. Instead, the broader trend of the presence or absence of cost savings in individual studies comparing reused and once-used SUMDs was reported. 

### Future research 

This systematic review identified explicit research gaps and areas for improvement in existing research practice in this field. Firstly, as no eligible life cycle assessment studies were identified, there is a clear gap in current understanding of the environmental impacts of reprocessing high risk SUMDs. 

Secondly, there is a need for high quality cost-effectiveness studies for both cardiac catheterisation and implantable cardiac devices studies. Consistent with the views of the French National Academies of Medicine, Pharmacy and Surgery [40], future economic evaluation studies should include those which consider cost-effectiveness from a health system perspective in order to account for costs associated with European regulatory reprocessing requirements e.g. expensive up-front costs such as staff training and new equipment or premises purchases. Relatedly, studies should examine the costs to health systems of the different regulatory options established by the 2017 EU medical device regulation. Furthermore, the GRADE assessment for indirect costs resulted in very low certainty that the finding would be repeated in future studies (Attachment 6). 

Thirdly, although implantable cardiac device studies followed patients up for an average of three years [26], [27], [28], the average follow up period for primary safety outcomes of cardiac catheterisation device studies was much shorter, from admission to discharge [30], [29] or 3 months [31], [32]. Although the estimated risk of prion disease is low given that studies involved interventions at sites distant from neurological exposure, it is vital that available longitudinal data are used to study any associations between SUMD reprocessing and onset of acquired prion diseases, which have long incubation periods [37].

Finally, to improve the quality of SUMD reprocessing research, as well as adequately describing reprocessing oversight and processes, researchers should ensure that studies are adequately powered to detect effects for primary and rare event outcomes e.g., major complications, which was lacking in the studies included in this review (Attachment 4 ). Both actions, as well as moving from observational to randomised controlled trial study designs or analysis of surveillance system data in countries where SUMD reprocessing is in place, and adhering to relevant study design reporting standards would likely improve our confidence in the safety outcomes reported (Attachment 6 ). When the proposed research is undertaken and reported as recommended, future systematic reviews on this topic should examine the association between “reprocessing oversight” and safety, cost-effectiveness and environmental impacts. This would provide additional important insight into how reprocessing should be implemented. 

## Conclusions

Overall, there is still insufficient good-quality evidence to establish the safety, cost-effectiveness, and environmental impacts of reusing catheterisation or implantable SUMDs. The volume and type of available evidence differs by device type, for instance, data are lacking for major complications for catheterisation devices and for long-term outcomes generally. High-quality randomised controlled trials, analyses of national surveillance systems, cost-effectiveness studies, and life cycle assessments which adequately describe devices and reprocessing (processes and oversight) are required in order to facilitate better comparison across devices and reprocessing contexts. 

## Notes

### Competing interests

The authors declare that they have no competing interests.

### Funding

This work was funded by the HRB Evidence Centre, which is funded by the Department of Health, Ireland. The Department of Health played no role in the design, conduct, analysis or interpretation of review findings. All authors’ time on the work was supported by the HRB Evidence Centre. 

### Acknowledgments

We extend our thanks to our colleagues at the Health Products Regulatory Authority, James McCarthy, Patrick Murphy, Dhanashree Gokhale, and Jennifer Roche, to Dr Karen McNamara at the Department of Health and to our Health Information and Quality Authority colleagues Dr Kieran Walsh and, formerly, Dr Paul Carty for their conceptual input during the design phase of this review. Several of our colleagues within the Health Research Board Evidence Centre also provided invaluable assistance with individual components of the review; specifically, Jean Long contributed to the review conception and early draft review and Dr Leila Keshtkar, Dr Áine Teahan, and Dr Annette Burns completed aspects of data preparation and analysis: data extraction; data presentation; quality appraisal; and GRADE assessment. Finally, we thank three peer reviewers who provided considered and valuable feedback on the full report, of which the present article forms a component of; Dr Francesco Tessarolo, Dr Lisa Leung, MRCP and Dr Mark Gallagher, MD FRCPI. 

### Authors’ ORCIDs

- McGrath N: https://orcid.org/0000-0002-7716-727
- Waldron C: https://orcid.org/0000-0002-0408-8543
- Farragher A: https://orcid.org/0009-0007-6338-2447
- Walsh C: https://orcid.org/0000-0001-6599-9562
- Polisena J: https://orcid.org/0009-0007-6338-2447

## Supplementary Material

Grey literature table and detailed search strategies

Review eligibility criteria

Review review outcome selection

Quality assessment

Meta-analysis feasibility assessment

Grading of Recommendations, Assessment, Development and Evaluations

## Figures and Tables

**Table 1:**
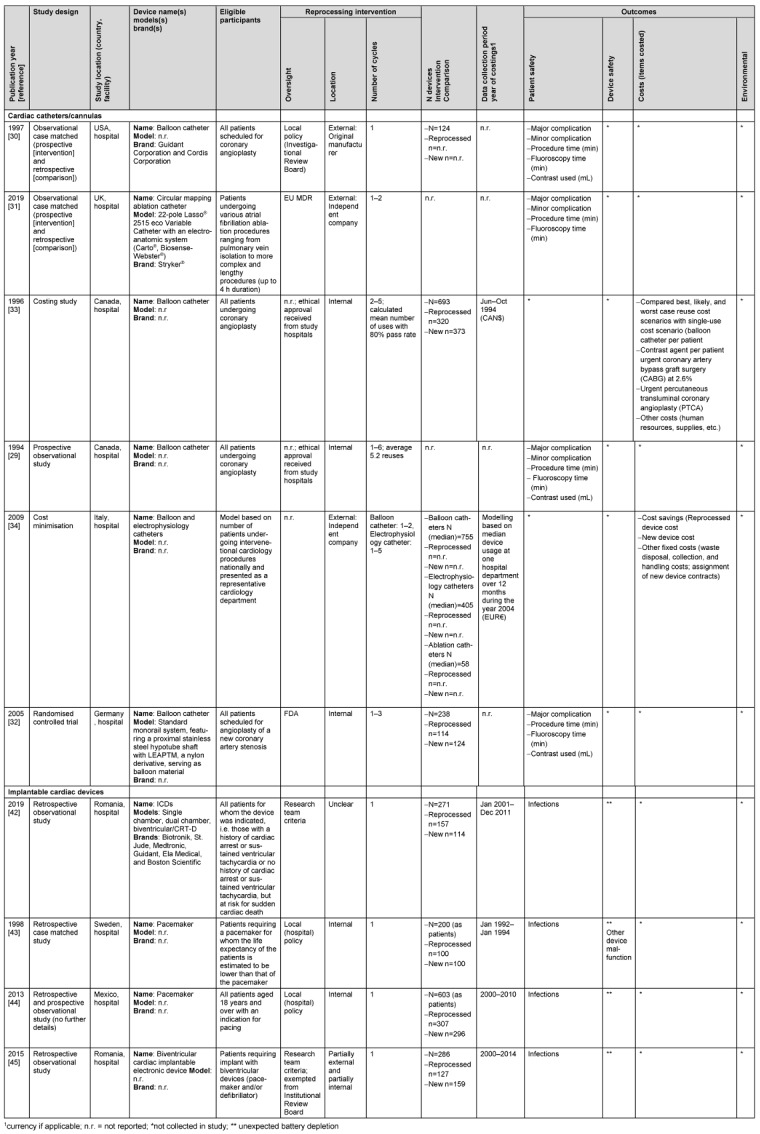
Characteristics of included studies

**Table 2:**
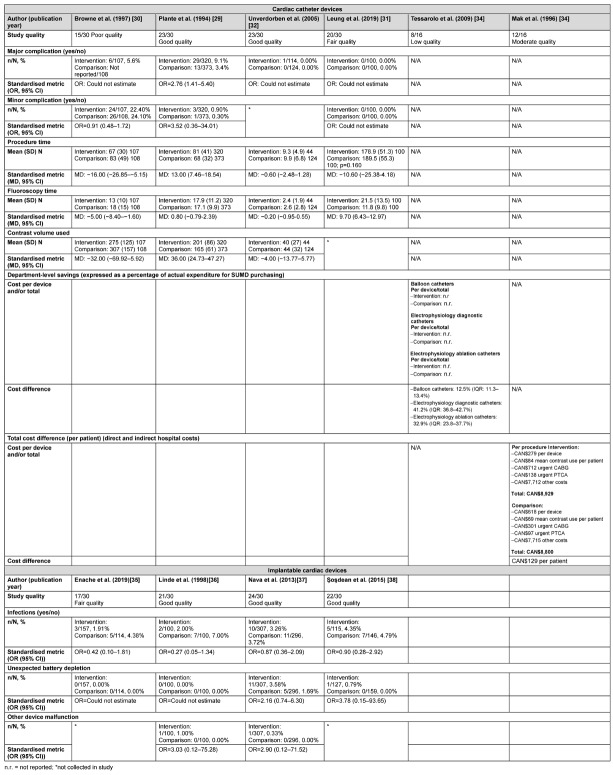
Narrative safety and cost outcomes for cardiac catheter and implantable cardiac devices
