# Supplementary material for: Safety, cost and environmental impact of reprocessing high risk single-use medical devices: a systematic review and meta-analysis
Source: GMS Hyg Infect Control. 2025 Jun 6;20:Doc25. doi: 10.3205/dgkh000554 (PMC12248001; doi:10.3205/dgkh000554)
Supplement: Grey literature table and detailed search strategies [file HIC-20-25-s-001.pdf]

## Attachment 1 Grey literature table and detailed search strategies

### Grey literature table

General scoping searches were carried out on the Google.com search engine to gain an initial idea of terminology and likely key terms. Reviewing literature in the area (retrieved from the Epistemonikos and Cochrane databases) also helped build up our search vocabulary. Initial search terms used included combinations of 'single-use device', 'SUD', 'SUMD', and 'single-use medical device', together with language around reprocessing and reuse. Further searches were carried out using the websites of relevant bodies (see Table S 1).

Table S 1 Websites included in supplementary grey literature search

| Organisation | Website |
|---|---|
| *Bundesgesundheitministerium* (Federal Ministry of Health, Germany) | https://www.bundesgesundheitsministerium.de/en/ministry/the-federal-ministry-of-health.html |
| Center for the Evaluation of Value and Risk in Health (Cost-Effectiveness Analysis (CEA) Registry, Tufts University, Boston, Massachusetts, United States of America (USA)) | https://cevr.tuftsmedicalcenter.org/databases/cea-registry |
| Centre for Reviews and Dissemination (CRD, University of York, United Kingdom (UK)) | https://www.york.ac.uk/crd/ |
| Competent Authorities for Medical Devices (CAMD) | https://www.camd-europe.eu/ |
| Department of Health, Ireland | https://www.gov.ie/en/organisation/department-of-health/# |
| European Commission | https://ec.europa.eu/info/index_en |
| European Union (EU) Law (Europa) | https://european-union.europa.eu/institutions-law-budget/law_en |
| European database on medical devices (EUDAMED) | https://ec.europa.eu/tools/eudamed/#/screen/home |
| Google | https://www.google.ie/ |
| International Health Technology Assessment Database | https://www.inahta.org/ |
| Health Systems Evidence | https://www.healthsystemsevidence.org/ |
| Lenus | https://www.lenus.ie/ |
| Livivo | https://www.livivo.de |
| MedTech Europe (trade association) | https://www.medtecheurope.org/ |
| OpenGrey repository | http://www.opengrey.eu |
| PROSPERO registry | https://www.crd.york.ac.uk/prospero/ |
| World Health Organization | https://www.who.int/ |

Attachment to: McGrath N, Waldron C, Farragher A, Walsh C, Polisena J. Safety, cost and environmental impact of reprocessing high risk single-use medical devices: a systematic review and meta-analysis. GMS Hyg Infect Control. 2025;20:Doc25. DOI: 10.3205/dgkh000554

## Search strategies

Table S 2 Overview of database results

| Database | Date of search | No. of results |
|---|---|---|
| **Ovid MEDLINE** | 25 July 2022 | 3,617 |
| **Embase** | 25 July 2022 | 2,079 |
| **Cochrane Trial Register** | 25 July 2022 | 291 |
| **Cochrane Library (John Wiley & Sons Inc.)** | 25 July 2022 | 3 |
| **Dimensions** | 25 July 2022 | 304 |
| **Total before deduplication** | | 6,294 |
| **Total after deduplication** | | 5,041 |
| **Total retained for analysis after screening** | Health setting | 18 |
| **Total retained for analysis after screening** | Laboratory setting | 33 |
| **Total added from reference chasing** | | 1 |

### Embase

Table S 3 Embase search strategy 25 July 2022

| Query number (#) | Search terms | Results |
|---|---|---|
| #46 | #45 AND ('article'/it OR 'article in press'/it OR 'chapter'/it OR 'conference abstract'/it OR 'conference paper'/it OR 'preprint'/it OR 'review'/it OR 'short survey'/it) | 2,079 |
| #45 | #30 AND #34 AND [1994-2022]/py | 2,171 |
| #44 | #30 AND #34 | 2,269 |
| #43 | #35 NOT #42 | 2,268 |
| #42 | 'single-use versus reusable medical devices in spinal fusion surgery':ti | 1 |
| #41 | #35 NOT #40 | 2,268 |
| #40 | 'a cost-effectiveness analysis of exalt model d single-use duodenoscope versus':ti | 1 |
| #39 | #35 NOT #38 | 2,268 |

| Query number (#) | Search terms | Results |
|---|---|---|
| #38 | 'economic analysis of reprocessing single-use medical devices':ti | 1 |
| #37 | #35 NOT #36 | 2,268 |
| #36 | 'systematic review of reusable versus disposable laparoscopic instruments':ti | 1 |
| #35 | #30 AND #34 | 2,269 |
| #34 | #31 OR #32 OR #33 | 11,845,373 |
| #33 | #21 OR #22 OR #23 OR #24 OR #25 OR #26 OR #27 OR #28 OR #29 | 9,248,690 |
| #32 | #14 OR #15 OR #16 OR #17 OR #18 OR #19 OR #20 | 2,212,471 |
| #31 | #5 OR #6 OR #7 OR #8 OR #9 OR #10 OR #11 OR #12 OR #13 | 1,396,789 |
| #30 | #1 OR #2 OR #3 OR #4 | 2,947 |
| #29 | (equipment NEAR/5 (safety OR risk OR infection OR adverse OR malfunction)):ab,ti,kw | 4,318 |
| #28 | (instrument NEAR/5 (safety OR risk OR infection OR adverse OR malfunction)):ab,ti,kw | 2,472 |
| #27 | (device* NEAR/5 (safety OR risk OR infection OR adverse OR malfunction)):ab,ti,kw | 22,199 |
| #26 | 'instrument sterilization'/exp OR 'disinfection'/exp OR disinfect*:ab,ti,kw OR sterilz*:ab,ti,kw OR sterilis*:ab,ti,kw OR quarantin*:ab,ti,kw | 89,657 |
| #25 | 'infection'/exp OR 'hygiene'/exp OR 'cross infection'/exp | 4,264,422 |
| #24 | 'risk'/exp | 2,850,294 |
| #23 | 'adverse event'/exp | 967,425 |
| #22 | 'safety procedure'/exp | 64,078 |

| Query number (#) | Search terms | Results |
|---|---|---|
| | | |
| #21 | 'safety'/exp OR safety:ab,ti,kw OR incidence:ab,ti,kw OR mortality:ab,ti,kw | 3,430,453 |
| #20 | 'environmental aspects and related phenomena'/exp | 2,075,373 |
| #19 | ('waste' NEAR/5 (dispos* OR management OR cost OR impact)):ab,ti,kw | 23,057 |
| #18 | (environment* NEAR/5 (dispos* OR management OR cost OR impact)):ab,ti,kw | 50,468 |
| #17 | (waste OR environment*) | 25,727 |
| #16 | 'environmental sustainability'/exp OR 'environmental impact'/exp | 95,197 |
| #15 | 'waste management'/exp | 213,588 |
| #14 | 'waste disposal'/exp | 19,39 |
| #13 | (cost NEAR/5 (benefit* OR effectiv* OR comparat* OR analy*)):ab,ti,kw | 278,703 |
| #12 | 'cost benefit':ab,ti,kw OR 'cost-benefit':ab,ti,kw OR 'cost analysis':ab,ti,kw OR 'cost-analysis':ab,ti,kw OR 'cost compar*':ab,ti,kw OR 'cost implication*':ab,ti,kw OR 'cost effectiv*' OR 'cost-effectiv*':ab,ti,kw OR cost:ab,ti,kw | 747,969 |
| #11 | 'cost minimization analysis'/exp | 3,797 |
| #10 | 'hospital cost'/exp | 43,215 |
| #9 | 'health economics'/exp | 987,523 |
| #8 | 'health care financing'/exp | 13,727 |
| #7 | 'health care cost'/exp | 323,080 |
| #6 | 'cost control'/exp | 73,496 |

| Query number (#) | Search terms | Results |
|---|---|---|
| #5 | 'cost benefit analysis'/exp | 90,954 |
| #4 | ((sud OR sumd) NEAR/20 (instrument* OR device* OR equipment)):ab,ti,kw | 146 |
| #3 | ('certified reprocessing' NEAR/20 (instrument* OR device* OR equipment)):ab,ti,kw | 2 |
| #2 | ((reuse OR 're-use' OR 're use' OR reprocess* OR remanufact*) NEAR/20 (instrument* OR device* OR equipment)):ab,ti,kw | 1,807 |
| #1 | ('single use' NEXT/10 (instrument* OR device* OR equipment)):ab,ti,kw | 1,276 |

**MEDLINE**

Table S 4 MEDLINE search strategy 25 July 2022

| Query number (#) | Search terms | Results |
|---|---|---|
| #1 | ((reuse or re-use or reusing or reprocess* or remanufact* or resterili#e*) adj10 (instrument* or device* or equipment)).mp. | 3,908 |
| #2 | (certified reproc* or sud or SUMD or (single adj use* adj10 (instrument* or device* or equipment))).mp. | 7,633 |
| #3 | or/1-2 | 11,259 |
| #4 | exp Equipment Reuse/ | 3,158 |
| #5 | exp "Equipment and Supplies"/ | 1,610,810 |
| #6 | ((medical or surgical) and (device* or instrument* or equipment)).mp. | 293,141 |
| #7 | (single adj use* adj10 (instrument* or device* or equipment)).mp. | 966 |
| #8 | exp Surgical Instruments/ | 25,809 |

| Query number (#) | Search terms | Results |
|---|---|---|
| #9 | exp Surgical Equipment/ | 286,785 |
| #10 | exp disposable equipment/ | 5,265 |
| #11 | exp "Equipment and Supplies, Hospital"/ | 27,237 |
| #12 | or/4-11 | 1,775,417 |
| #13 | exp Cost-Benefit Analysis/ | 90,518 |
| #14 | exp "Cost Control"/ | 34,037 |
| #15 | health care cost effectiveness.mp. | 15 |
| #16 | exp Healthcare Financing/ | 1,202 |
| #17 | exp "Health Care Economics and Organizations"/ | 1,642,271 |
| #18 | exp Hospital Costs/ | 11,865 |
| #19 | " cost minimi*ation analysis".mp. | 808 |
| #20 | ('cost benefit' or 'cost-benefit' or 'cost analysis' or 'cost-analysis' or 'cost compar*' or 'cost implication*' or 'cost-effectiv*' or cost).mp. | 615,324 |
| #21 | (cost adj5 (benefit* or effectiv* or comparat* or analy*)).mp. | 280,595 |
| #22 | or/13-21 | 2,004,556 |
| #23 | exp Carbon Footprint/ | 903 |
| #24 | (waste adj5 (dispos* or management or cost or impact)).mp. | 56,496 |
| #25 | (environment* adj5 (dispos* or management or cost or impact)).mp. | 42,189 |
| #26 | ((environment* adj5 sustainab*) or carbon footprint or recycl*).mp. | 72,242 |
| #27 | or/23-26 | 158,865 |
| #28 | exp Safety Management/ or Safety Management.mp. | 22,460 |

Attachment to: McGrath N, Waldron C, Farragher A, Walsh C, Polisena J. Safety, cost and environmental impact of reprocessing high risk single-use medical devices: a systematic review and meta-analysis. GMS Hyg Infect Control. 2025;20:Doc25. DOI: 10.3205/dgkh000554

| Query number (#) | Search terms | Results |
|---|---|---|
| #29 | exp Patient Safety/ | 24,697 |
| #30 | exp Equipment Safety/ | 10,437 |
| #31 | ((device or equipment) adj5 (defect* or failure* or misuse or malfunction*)).mp. | 66,479 |
| #32 | ((contaminat* or complicat* or erosion) adj5 (instrument* or device* or equipment)).mp. | 29,419 |
| #33 | (safety or incidence or mortality).mp. | 2,718,406 |
| #34 | (Adverse event or Adverse Event Outcome).mp. | 34,945 |
| #35 | exp "Risk Evaluation and Mitigation"/ | 64 |
| #36 | exp Risk/ | 1,350,136 |
| #37 | (Infection Control or Infection, Healthcare Associated).mp. | 47,963 |
| #38 | exp Hygiene/ or exp Cross Infection/ | 104,935 |
| #39 | exp instrument sterilization/ or exp disinfection/ or (disinfect* or sterilz* or sterilis* or quarantin*).mp. | 70,970 |
| #40 | exp Sterilization/ or (sterili* adj5 (instrument* or device* or equipment)).mp. | 34,448 |
| #41 | or/28-40 | 3,867,074 |
| #42 | (substance adj5 disorder*).mp. | 117,748 |
| #43 | 22 or 27 or 41 | 5,675,733 |
| #44 | 3 and 43 | 5,423 |
| #45 | limit 44 to yr="1994 -Current" | 5,203 |
| #46 | limit 45 to (comment or editorial or letter or newspaper article) | 276 |
| #47 | 45 not 46 | 4,927 |
| #48 | "substance use".ti,ab,kw. | 45,398 |

| Query number (#) | Search terms | Results |
|---|---|---|
| #49 | 47 not 48 | 3,617 |

Database(s): **Ovid MEDLINE and Epub Ahead of Print, In-Process, In-Data-Review and Other Non-Indexed Citations, Daily and Versions** 1946 to July 25, 2022

**Dimensions database**

Table S 5 Dimensions database search strategy 25 July 2022

| Search term | Results |
|---|---|
| ("reprocessing device"~5) OR ("reuse device" ~5) OR ("resterili?e device"~5)<br><br>Filter: Title and Abstract search | 304 |

**Cochrane Library and Register**

Table S 6 Cochrane Library (John Wiley & Sons Inc.) search strategy 25 July 2022

| Query number (#) | Search terms | Results |
|---|---|---|
| #1 | (reuse OR re-use or reprocess* or remanufact*) and ((SUD or SUMD* or single-use or "single use") NEAR/5 (device* or instrument* or equipment)) | 14 |
| #2 | MeSH descriptor: [Equipment and Supplies] explode all trees | 53,231 |
| #3 | MeSH descriptor: [Equipment Reuse] this term only | 101 |
| #4 | MeSH descriptor: [Equipment and Supplies] this term only | 186 |
| #5 | [mh "Equipment and Supplies"[mj]] | 8,347 |
| #6 | #1 or #4 or #3 | 294 |

**References (supplemental files)**


1 Deeks JJ, Dinnes J, Abdulnabi R, *et al.* Evaluating non-randomised intervention studies. *Health Technol Assess Winch Engl* 2003;**7**:iii–x, 1–173. doi:https://doi.org/10.3310/hta7270

2 Hooper P, Jutai JW, Strong G, *et al.* Age-related macular degeneration and low-vision rehabilitation: a systematic review. *Can J Ophthalmol J Can Ophtalmol* 2008;**43**:180–7. doi:https://doi.org/10.3129/i08-001

3 Evers S, Goossens M, de Vet H, *et al.* Criteria list for assessment of methodological quality of economic evaluations: Consensus on Health Economic Criteria. *Int J Technol Assess Health Care* 2005;**21**:240–5. doi:https://doi.org/

4 Jacobs P, Polisena J, Hailey D, *et al.* Economic analysis of reprocessing single-use medical devices: a systematic literature review. *Infect Control Hosp Epidemiol* 2008;**29**:297–301. doi:https://doi.org/10.1086/529587

5 Hamberg-van Reenen HH, Proper KI, van den Berg M. Worksite mental health interventions: a systematic review of economic evaluations. *Occup Environ Med* 2012;**69**:837–45. doi:https://doi.org/10.1136/oemed-2012-100668

6 van Dongen J, Proper K, van Wier MF, *et al.* Systematic review on the financial return of worksite health promotion programmes aimed at improving nutrition and/or increasing physical activity. *Obes Rev Off J Int Assoc Study Obes* 2011;**12**:1031–49. doi:https://doi.org/10.1111/j.1467-789X.2011.00925.x

7 Uegaki K, de Bruijne MC, Lambeek L, *et al.* Economic evaluations of occupational health interventions from a corporate perspective - a systematic review of methodological quality. *Scand J Work Environ Health* 2010;**36**:273–88. doi:https://doi.org/10.5271/sjweh.3017

8 Browne K, Maldonado R, Telatnik M, *et al.* Initial experience with reuse of coronary angioplasty catheters in the United States. *J Am Coll Cardiol* 1997;**30**:1735–40. doi:https://doi.org/10.1016/S0735-1097(97)00362-8

9 Leung L, Evranos B, Grimster A, *et al.* Remanufactured circular mapping catheters: safety, effectiveness and cost. *J Interv Card Electrophysiol* 2019;**56**:205–11. doi:https://doi.org/10.1007/s10840-018-0497-x

10 Plante S, Strauss BH, Goulet G, *et al.* Reuse of balloon catheters for coronary angioplasty: A potential cost-saving strategy? *J Am Coll Cardiol* 1994;**24**:1475–81. doi:https://doi.org/10.1016/0735-1097(94)90142-2

11 Unverdorben M, Degenhardt R, Erny D, *et al.* Clinical and angiographic procedural and mid-term outcome with new versus reused balloon catheters in percutaneous coronary interventions. *Indian Heart J* 2005;**57**:114–20. doi:https://doi.org/

12 Enache B, Șoșdean R, Macarie R, *et al.* Assessing the safety of implantable cardioverter-defibrillator reuse—A retrospective case-control study. *PACE - Pacing Clin Electrophysiol* Published Online First: 2019. doi:https://doi.org/10.1111/pace.13742

13 Linde C, Bocray A, Jonsson H. Re-used pacemakers: as safe as new? A retrospective case-control study. *Eur Heart J* 1998;**19**:154–7. doi:https://doi.org/10.1053/euhj.1997.0728

14 Nava S, Morales J, Márquez M, *et al.* Reuse of pacemakers: comparison of short and long-term performance. *Circulation* 2013;**127**:1177-1183. doi:https://doi.org/10.1161/CIRCULATIONAHA.113.001584

15 Şoşdean R, Mornoş C, Enache B, *et al.* Safety and feasibility of biventricular devices reuse in general and elderly population – A single-center retrospective cohort study. *Clin Interv Aging* 2015;**10**:1311–8. doi:https://doi.org/10.2147/CIA.S88805

16 Tessarolo F, Disertori M, Guarrera GM, *et al.* Reprocessing single-use cardiac catheters for interventional cardiology. A cost-minimization model for estimating potential saving at departmental scale and national level. *Ital J Public Health* 2009;**6**:140–9.

17 Mak K-H, Eisenberg MJ, Eccleston DS, *et al.* Cost-efficacy modeling of catheter reuse for percutaneous transluminal coronary angioplasty. *J Am Coll Cardiol* 1996;**28**:106–11. doi:https://doi.org/10.1016/0735-1097(96)00097-6
