# Supplementary material for: Safety, cost and environmental impact of reprocessing high risk single-use medical devices: a systematic review and meta-analysis
Source: GMS Hyg Infect Control. 2025 Jun 6;20:Doc25. doi: 10.3205/dgkh000554 (PMC12248001; doi:10.3205/dgkh000554)
Supplement: Review eligibility criteria [file HIC-20-25-s-002.pdf]

Attachment 2 Review eligibility criteria

Table S 1 Inclusion and exclusion criteria using population, intervention, comparator, and outcome(s) (PICO) and other relevant criteria

Element	Inclusion	Exclusion
Population	Single-use medical devices, or purpose-built components thereof, contaminated from clinical use on human patients or artificially using human bacteria, viruses, etc.	Single-use medical devices contaminated from clinical use in non-human patients.
Intervention	A newly developed or established reprocessing method which involved device cleaning, disinfection, sterilisation, or related procedures, and device function and safety testing.<br>Contaminated devices were exposed to one or more reprocessing cycles.	Reprocessing of reusable medical devices.<br>Reprocessing of single-use components of otherwise reusable medical devices.<br>It is unclear whether the reprocessing involved both the cleaning and related procedures as well as the function and safety testing aspects.<br>For studies with multiple reuse cycles, devices reused on the same person (i.e. single-person reuse).
Comparator	Unused (i.e. new) SUDs.<br>Manufacturer specifications for device sterilisation, safety, and functioning.	Reusable device alternative of a single-use medical device (e.g. the same device made from different materials).<br>Contaminated devices which have not yet been reprocessed.
Outcome(s)	Device function and safety: Device sterility, device degradation, device failure, device corrosion, or other device-specific reprocessing process-related function and safety outcomes.<br>Environmental impact: Environmental and human health impacts.<br>Environmental impacts include carbon emissions for new device production and reprocessing, disposal waste volume, and other environmental impacts. Human health impacts include human health effects of air pollution, human health effects of chemical exposure e.g., cancer, breathing issues.<br>Cost: First use device purchase cost, SUD reprocessing cost, SUD disposal cost, and costs associated with safety and environmental outcomes.	Does not provide data for all reprocessing components (i.e. device cleaning/sterilisation and device safety and functioning testing).

Supplemental file 2 Review eligibility criteria

Table S 1 Inclusion and exclusion criteria using population, intervention, comparator, and outcome(s) (PICO) and other relevant criteria

Element	Inclusion	Exclusion
Population	Single-use medical devices, or purpose-built components thereof, contaminated from clinical use on human patients or artificially using human bacteria, viruses, etc.	Single-use medical devices contaminated from clinical use in non-human patients.
Intervention	A newly developed or established reprocessing method which involved device cleaning, disinfection, sterilisation, or related procedures, and device function and safety testing.<br>Contaminated devices were exposed to one or more reprocessing cycles.	Reprocessing of reusable medical devices.<br>Reprocessing of single-use components of otherwise reusable medical devices.<br>It is unclear whether the reprocessing involved both the cleaning and related procedures as well as the function and safety testing aspects.<br>For studies with multiple reuse cycles, devices reused on the same person (i.e. single-person reuse).
Comparator	Unused (i.e. new) SUDs.<br>Manufacturer specifications for device sterilisation, safety, and functioning.	Reusable device alternative of a single-use medical device (e.g. the same device made from different materials).<br>Contaminated devices which have not yet been reprocessed.
Outcome(s)	Device function and safety: Device sterility, device degradation, device failure, device corrosion, or other device-specific reprocessing process-related function and safety outcomes.<br>Environmental impact: Environmental and human health impacts.<br>Environmental impacts include carbon emissions for new device production and reprocessing, disposal waste volume, and other environmental impacts. Human health impacts include human health effects of air pollution, human health effects of chemical exposure e.g., cancer, breathing issues.<br>Cost: First use device purchase cost, SUD reprocessing cost, SUD disposal cost, and costs associated with safety and environmental outcomes.	Does not provide data for all reprocessing components (i.e. device cleaning/sterilisation and device safety and functioning testing).

Attachment to: McGrath N, Waldron C, Farragher A, Walsh C, Polisena J. Safety, cost and environmental impact of reprocessing high risk single-use medical devices: a systematic review and meta-analysis. GMS Hyg Infect Control. 2025;20:Doc25. DOI: 10.3205/dgkh000554

Element	Inclusion	Exclusion
Study design	In vitro primary studies.	Conference abstracts<br>Qualitative studies<br>Case reports or series<br>Ecological studies<br>Studies which do not describe a methodology (e.g. literature reviews)<br>Systematic reviews
Language	English, German.	Any other language.

References (supplemental files)

1 Deeks JJ, Dinnes J, Abdulnabi R, *et al.* Evaluating non-randomised intervention studies. *Health Technol Assess Winch Engl* 2003;**7**:iii–x, 1–173. doi:https://doi.org/10.3310/hta7270

2 Hooper P, Jutai JW, Strong G, *et al.* Age-related macular degeneration and low-vision rehabilitation: a systematic review. *Can J Ophthalmol J Can Ophtalmol* 2008;**43**:180–7. doi:https://doi.org/10.3129/i08-001

3 Evers S, Goossens M, de Vet H, *et al.* Criteria list for assessment of methodological quality of economic evaluations: Consensus on Health Economic Criteria. *Int J Technol Assess Health Care* 2005;**21**:240–5. doi:https://doi.org/

4 Jacobs P, Polisena J, Hailey D, *et al.* Economic analysis of reprocessing single-use medical devices: a systematic literature review. *Infect Control Hosp Epidemiol* 2008;**29**:297–301. doi:https://doi.org/10.1086/529587

5 Hamberg-van Reenen HH, Proper KI, van den Berg M. Worksite mental health interventions: a systematic review of economic evaluations. *Occup Environ Med* 2012;**69**:837–45. doi:https://doi.org/10.1136/oemed-2012-100668

6 van Dongen J, Proper K, van Wier MF, *et al.* Systematic review on the financial return of worksite health promotion programmes aimed at improving nutrition and/or increasing physical activity. *Obes Rev Off J Int Assoc Study Obes* 2011;**12**:1031–49. doi:https://doi.org/10.1111/j.1467-789X.2011.00925.x

7 Uegaki K, de Bruijne MC, Lambeek L, *et al.* Economic evaluations of occupational health interventions from a corporate perspective - a systematic review of methodological quality. *Scand J Work Environ Health* 2010;**36**:273–88. doi:https://doi.org/10.5271/sjweh.3017

8 Browne K, Maldonado R, Telatnik M, *et al.* Initial experience with reuse of coronary angioplasty catheters in the United States. *J Am Coll Cardiol* 1997;**30**:1735–40. doi:https://doi.org/10.1016/S0735-1097(97)00362-8

9 Leung L, Evranos B, Grimster A, *et al.* Remanufactured circular mapping catheters: safety, effectiveness and cost. *J Interv Card Electrophysiol* 2019;**56**:205–11. doi:https://doi.org/10.1007/s10840-018-0497-x

10 Plante S, Strauss BH, Goulet G, *et al.* Reuse of balloon catheters for coronary angioplasty: A potential cost-saving strategy? *J Am Coll Cardiol* 1994;**24**:1475–81. doi:https://doi.org/10.1016/0735-1097(94)90142-2

11 Unverdorben M, Degenhardt R, Erny D, *et al.* Clinical and angiographic procedural and mid-term outcome with new versus reused balloon catheters in percutaneous coronary interventions. *Indian Heart J* 2005;**57**:114–20. doi:https://doi.org/

12 Enache B, Șoșdean R, Macarie R, *et al.* Assessing the safety of implantable cardioverter-defibrillator reuse—A retrospective case-control study. *PACE - Pacing Clin Electrophysiol* Published Online First: 2019. doi:https://doi.org/10.1111/pace.13742

13 Linde C, Bocray A, Jonsson H. Re-used pacemakers: as safe as new? A retrospective case-control study. *Eur Heart J* 1998;**19**:154–7. doi:https://doi.org/10.1053/euhj.1997.0728

14 Nava S, Morales J, Márquez M, *et al.* Reuse of pacemakers: comparison of short and long-term performance. *Circulation* 2013;**127**:1177-1183. doi:https://doi.org/10.1161/CIRCULATIONAHA.113.001584

15 Şoşdean R, Mornoş C, Enache B, *et al.* Safety and feasibility of biventricular devices reuse in general and elderly population – A single-center retrospective cohort study. *Clin Interv Aging* 2015;**10**:1311–8. doi:https://doi.org/10.2147/CIA.S88805

16 Tessarolo F, Disertori M, Guarrera GM, *et al.* Reprocessing single-use cardiac catheters for interventional cardiology. A cost-minimization model for estimating potential saving at departmental scale and national level. *Ital J Public Health* 2009;**6**:140–9.

17 Mak K-H, Eisenberg MJ, Eccleston DS, *et al.* Cost-efficacy modeling of catheter reuse for percutaneous transluminal coronary angioplasty. *J Am Coll Cardiol* 1996;**28**:106–11. doi:https://doi.org/10.1016/0735-1097(96)00097-6
