# Supplementary material for: Safety, cost and environmental impact of reprocessing high risk single-use medical devices: a systematic review and meta-analysis
Source: GMS Hyg Infect Control. 2025 Jun 6;20:Doc25. doi: 10.3205/dgkh000554 (PMC12248001; doi:10.3205/dgkh000554)
Supplement: Review review outcome selection [file HIC-20-25-s-003.pdf]

Attachment 3 Review review outcome selection

Table S 1 shows the full spectrum of outcomes collected in eligible studies. Items in red font denote studies or individual outcomes excluded at this stage. We excluded studies which did not report on patient safety or only provided patient or device safety outcomes via subjective measurement methods (e.g. surgeon opinion). For many outcomes, we provided a note (highlighted in yellow) indicating a possible strategy of combining and grouping outcomes. This is not indicative of finalised outcome grouping decisions. One study was excluded during this outcome selection process.

Table S 1 Safety outcome selection and preliminary groupings

Author	Safety outcome name	Outcome type	Definition and note on preliminary grouping
**Cardiac catheters**			
Browne *et al.* (1997) [115]	Crossing success	Device function (direct)	Opposite to crossing failure in Plante *et al.* (1994)
	Pyrogen reactions	Patient safety (direct)	Temperature and white blood cell (WBC) count<br>MINOR COMPLICATION
	Evidence of subsequent myocardial infarction (MI) or requirement for emergent percutaneous or surgical revascularisation of the target vessel	Patient safety (direct)	All patients were followed until hospital discharge for evidence of subsequent MI or requirement for emergent percutaneous or surgical revascularisation of the target vessel.<br>MAJOR COMPLICATION
	Procedure time	Patient safety (indirect)	As name
	Fluoroscopy time	Patient safety (indirect)	As name
	Dye volume	Patient safety (indirect)	As name
	Number of balloons used per lesion	Device function (indirect)	Before and after crossing
	Death	Patient safety (direct)	As name<br>MAJOR COMPLICATION
Hoffman *et al.*	Pushability	Device function (direct)	Pushability of Intravascular ultrasound catheter (IVUS) (subjective measure)

Attachment to: McGrath N, Waldron C, Farragher A, Walsh C, Polisena J. Safety, cost and environmental impact of reprocessing high risk single-use medical devices: a systematic review and meta-analysis. GMS Hyg Infect Control. 2025;20:Doc25. DOI: 10.3205/dgkh000554

Author	Safety outcome name	Outcome type	Definition and note on preliminary grouping
(2000) [134]	Trackability	Device function (direct)	Trackability of IVUS catheter (subjective measure)
	Ease of moving the IVUS catheter on the guide wire	Device function (direct)	Ease of moving on guide wire (subjective measure)
	Imaging failure	Device function (direct)	Lesion could be reached (early failure) and imaged
	Near-field image quality	Device function (direct)	Characteristic of image quality
	Far-field image quality	Device function (direct)	Characteristic of image quality
	Ring-down artefact	Device function (direct)	Characteristic of image quality
	Image homogeneity	Device function (direct)	Characteristic of image quality
	4. Procedure duration (Paroxysmal atrial fibrillation, Persistent atrial fibrillation, re-do cases)	Patient safety (indirect)	As name
	5. Fluoroscopy duration by pulmonary vein isolation only, or pulmonary vein isolation + other	Patient safety (indirect)	As name
Leung *et al.* (2019) [116]	3. Patient major complication	Patient safety (direct)	Complications that did not have any likely relationship to the catheter were also recorded up until the point of discharge from hospital, including any major adverse cardiovascular/cerebrovascular events (MACCEs), vascular injury, or cardiac tamponade. Medical records were reviewed for evidence of complications of the procedure occurring in the period within 3 months after ablation, and for any pyrexial or infective illness reported in this period. MAJOR COMPLICATION

Author	Safety outcome name	Outcome type	Definition and note on preliminary grouping
	2. Patient minor complication	Patient safety (direct)	Medical records were reviewed for evidence of complications of the procedure occurring in the period within 3 months after ablation, and for any pyrexial or infective illness reported in this period. MINOR COMPLICATION
	Mapping catheter failure	Device function (direct)	Failure of communication with the electro-anatomic mapping system
	Other catheter failure	Device function (indirect)	Physical defect or deformation of the catheter on inspection after use (subjective measure)
Plante *et al.* (1994) [118]	Angiography success	Device function (indirect)	A lesional residual stenosis <50%, as determined by visual assessment
	Clinical success	Patient safety and device function	Angiographically successful angioplasty of all attempted lesions without in-hospital adverse clinical event, defined as death, MI, stroke, emergency angioplasty, or bypass surgery. COMBINE WITH MAJOR COMPLICATION IF POSSIBLE
	Clinical failure	Device function (direct)	If all attempted lesions could not be dilated successfully CROSSING FAILURE
	4. Procedure duration	Patient safety (indirect)	As name
	5. Fluoroscopy time	Patient safety (indirect)	As name
	6. Volume of contrast medium used	Patient safety (indirect)	As name
	7. The number of catheters required per lesion	Device function (indirect)	As name
	2. Fever: temperatures, creatine kinase (CK) levels	Patient safety (direct)	Temperature was >38 °C buccal or >38.5 °C rectal MINOR COMPLICATION

Author	Safety outcome name	Outcome type	Definition and note on preliminary grouping
	Length of hospital stay	Patient safety (indirect)	As name
Unverdorben *et al.* (2005) [120]	Device (balloon catheter) success	Device function (direct)	Crossing of the lesion with balloon and inflation of the balloon within the lesion
	19. Procedure success	Device function (indirect)	A residual stenosis of <30%, achieved either by stand-alone balloon angioplasty, stenting, or by another means<br>Angiographic success in Plante *et al.* (1994)
	3.Complications (thrombus; acute and subacute MI)	Patient safety (direct)	A thrombus was defined as a non-calcified filling defect within the vascular lumen, which was visible in several views and which could migrate to the peripheral artery. An acute thrombosis was defined by a total occlusion (Transient myocardial ischemia grade O) occurring within 24 hours of stent deployment whereas subacute thrombosis was the one that occurred >24 hours after stenting and <1 month after stenting. Q-wave MI was diagnosed with the occurrence of new Q-waves (>0.04 seconds) and rise of CK twice the upper limit of normal with significant increase in creatine phosphokinase-isoenzyme levels (CK-MB), whereas in non-Q-wave MIs, pathological Q-waves were absent.<br>MAJOR COMPLICATION
	Target lesion revascularisation rate	Device function (indirect)	Not reported
	Restenosis rate	Device function (indirect)	Not reported
	Late loss index	Device function (indirect)	The ratio between late loss and acute gain

Author	Safety outcome name	Outcome type	Definition and note on preliminary grouping
	7. The number of balloons used per procedure	Device function (indirect)	As name
	6. Consumption of contrast	Patient safety (indirect)	As name – as dye volume in Browne *et al.* (1997)
	Time taken for procedure	Patient safety (indirect)	As name
	Exposure time to radiation	Patient safety (indirect)	As fluoroscopy time in Plante *et al.* (1994), Browne *et al.* (1997) and Leung *et al.* (2019)
**Implantable cardiac devices**			
Enache *et al.* (2019) [111]	Complications: infections	Patient safety (direct)	Complications were defined as infections that required reintervention. COMPLICATIONS – combine
	2. + 3. Complications: device malfunction and replacements	Device function (direct)	Device malfunction and replacements due to untimely or unexpected battery depletion COMPLICATIONS (DEVICE) – combine
Linde *et al.* (1998) [112]	Complications rate: infections	Patient safety (direct)	Infections that required antibiotics and/or reoperations COMPLICATIONS – combine
	Complications: malfunction	Device function (direct)	Suspicion of pacemaker malfunction described in the file or causing replacement COMPLICATIONS (DEVICE) – combine
	Complications: replacements	Device function (direct)	Replacements due to battery depletion COMPLICATIONS (DEVICE) – combine
Nava *et al.* (2013) [113]	Unexpected battery depletion	Device function (direct)	For new pacemakers, early battery depletion was defined as depletion before the 6th year after implantation without relation to high pacing outputs or abnormal electrode impedances that would void the device warranty. Premature battery depletion was considered to have occurred when the elective replacement indication was reached between the 6th and 8th years after the initial implantation. The expected battery depletion in reused

Author	Safety outcome name	Outcome type	Definition and note on preliminary grouping
			devices would occur after the 4th year, early battery depletion would occur before the 2nd year, and premature battery depletion would occur between the 2nd and 4th years. BATTERY DEPLETION (DEVICE) – combine
	Infection	Patient safety (direct)	Four types of infection: 1) right endocarditis with electrode involvement; 2) sepsis without evidence of involvement of the circuit or pocket; 3) infection of the pacemaker pocket; and 4) extrusion of wires or generator. COMPLICATIONS – combine
	Malfunction	Device function (direct)	Device or electrode malfunction (software or hardware malfunction) COMPLICATIONS (DEVICE) – combine
Şoşdean *et al.* (2015) [114]	Device-related infection	Patient safety (direct)	As name COMPLICATIONS – combine
	4. Early battery depletion	Device function (direct)	As name BATTERY DEPLETION (DEVICE) – combine
	3. Device malfunction requiring reintervention	Device function (direct)	As name COMPLICATIONS (DEVICE) – combine
	5. Infection-related burden in 'elderly' and 'young' patients	Patient safety (direct)	As name

We also reviewed studies contributing cost data to determine the eligibility of available outcome data for this review. Table S 2 reports the criteria used by Health Research Board (HRB) reviewers (ÁT and NMG) to determine the eligibility of cost outcomes, and our final decisions on same.

Table S 2 Selection of cost outcomes

Author (Year)	Transparent methods	Actual costs used	Costing source	Other comments	Findings	HRB inclusion decision
Browne *et al.* (1997) [115]	No	No	Invoices	Cost savings are speculation	It is expected that the restoration process used in this study would permit institutions to save	Reject

Author (Year)	Transparent methods	Actual costs used	Costing source	Other comments	Findings	HRB inclusion decision
					40% of the original invoice cost of the product to the hospital.	
Leung *et al.* (2019) [116]	No	Cannot tell	List prices	Cannot tell what has actually been included in costing	Based on list prices, we have calculated the cost savings to our department arising from these 100 cases at GB£30,444 (Great British pounds).	Reject
Linde *et al.* (1998) [112]	No	No	Estimated cost – no further detail		The corresponding cost for the 317 reused units was US$31,700. This amounts to an estimated national savings of US$919,300.	Reject
Plante *et al.* (1994) [118]	No	Yes<br><br>Device cost only	Estimated reuse cost<br><br>New device cost source not reported	The additional costs associated with in-hospital adverse events (e.g. increased rates of bypass surgery and myocardial infarction, and prolonged procedure time and hospital stay) may be offsetting.	This study demonstrated important catheter cost differences between the reuse and single use centres. There was an estimated savings of CAN$110,000 over the 10-month course of the study in the reuse centres, which had an average of 5.2 balloon catheter reuses.	Inclined to reject – estimated reuse cost, estimated hospital savings

References (supplemental files)

1 Deeks JJ, Dinnes J, Abdulnabi R, *et al.* Evaluating non-randomised intervention studies. *Health Technol Assess Winch Engl* 2003;**7**:iii–x, 1–173. doi:https://doi.org/10.3310/hta7270

2 Hooper P, Jutai JW, Strong G, *et al.* Age-related macular degeneration and low-vision rehabilitation: a systematic review. *Can J Ophthalmol J Can Ophtalmol* 2008;**43**:180–7. doi:https://doi.org/10.3129/i08-001

3 Evers S, Goossens M, de Vet H, *et al.* Criteria list for assessment of methodological quality of economic evaluations: Consensus on Health Economic Criteria. *Int J Technol Assess Health Care* 2005;**21**:240–5. doi:https://doi.org/

4 Jacobs P, Polisena J, Hailey D, *et al.* Economic analysis of reprocessing single-use medical devices: a systematic literature review. *Infect Control Hosp Epidemiol* 2008;**29**:297–301. doi:https://doi.org/10.1086/529587

5 Hamberg-van Reenen HH, Proper KI, van den Berg M. Worksite mental health interventions: a systematic review of economic evaluations. *Occup Environ Med* 2012;**69**:837–45. doi:https://doi.org/10.1136/oemed-2012-100668

6 van Dongen J, Proper K, van Wier MF, *et al.* Systematic review on the financial return of worksite health promotion programmes aimed at improving nutrition and/or increasing physical activity. *Obes Rev Off J Int Assoc Study Obes* 2011;**12**:1031–49. doi:https://doi.org/10.1111/j.1467-789X.2011.00925.x

7 Uegaki K, de Bruijne MC, Lambeek L, *et al.* Economic evaluations of occupational health interventions from a corporate perspective - a systematic review of methodological quality. *Scand J Work Environ Health* 2010;**36**:273–88. doi:https://doi.org/10.5271/sjweh.3017

8 Browne K, Maldonado R, Telatnik M, *et al.* Initial experience with reuse of coronary angioplasty catheters in the United States. *J Am Coll Cardiol* 1997;**30**:1735–40. doi:https://doi.org/10.1016/S0735-1097(97)00362-8

9 Leung L, Evranos B, Grimster A, *et al.* Remanufactured circular mapping catheters: safety, effectiveness and cost. *J Interv Card Electrophysiol* 2019;**56**:205–11. doi:https://doi.org/10.1007/s10840-018-0497-x

10 Plante S, Strauss BH, Goulet G, *et al.* Reuse of balloon catheters for coronary angioplasty: A potential cost-saving strategy? *J Am Coll Cardiol* 1994;**24**:1475–81. doi:https://doi.org/10.1016/0735-1097(94)90142-2

11 Unverdorben M, Degenhardt R, Erny D, *et al.* Clinical and angiographic procedural and mid-term outcome with new versus reused balloon catheters in percutaneous coronary interventions. *Indian Heart J* 2005;**57**:114–20. doi:https://doi.org/

12 Enache B, Șoșdean R, Macarie R, *et al.* Assessing the safety of implantable cardioverter-defibrillator reuse—A retrospective case-control study. *PACE - Pacing Clin Electrophysiol* Published Online First: 2019. doi:https://doi.org/10.1111/pace.13742

13 Linde C, Bocray A, Jonsson H. Re-used pacemakers: as safe as new? A retrospective case-control study. *Eur Heart J* 1998;**19**:154–7. doi:https://doi.org/10.1053/euhj.1997.0728

14 Nava S, Morales J, Márquez M, *et al.* Reuse of pacemakers: comparison of short and long-term performance. *Circulation* 2013;**127**:1177-1183. doi:https://doi.org/10.1161/CIRCULATIONAHA.113.001584

15 Şoşdean R, Mornoş C, Enache B, *et al.* Safety and feasibility of biventricular devices reuse in general and elderly population – A single-center retrospective cohort study. *Clin Interv Aging* 2015;**10**:1311–8. doi:https://doi.org/10.2147/CIA.S88805

16 Tessarolo F, Disertori M, Guarrera GM, *et al.* Reprocessing single-use cardiac catheters for interventional cardiology. A cost-minimization model for estimating potential saving at departmental scale and national level. *Ital J Public Health* 2009;**6**:140–9.

17 Mak K-H, Eisenberg MJ, Eccleston DS, *et al.* Cost-efficacy modeling of catheter reuse for percutaneous transluminal coronary angioplasty. *J Am Coll Cardiol* 1996;**28**:106–11. doi:https://doi.org/10.1016/0735-1097(96)00097-6
