# Supplementary material for: Safety, cost and environmental impact of reprocessing high risk single-use medical devices: a systematic review and meta-analysis
Source: GMS Hyg Infect Control. 2025 Jun 6;20:Doc25. doi: 10.3205/dgkh000554 (PMC12248001; doi:10.3205/dgkh000554)
Supplement: Quality assessment [file HIC-20-25-s-004.pdf]

Attachment 4 Quality assessment

Details of adaptations to the Downs and Black checklist

The original Downs and Black checklist consists of 27 items across the following methodological components: reporting, external validity, internal validity (bias and confounding), and power. Twenty-six items were rated either as yes (1) or no/unable to determine (0), and one item was rated on a 3-point scale (yes=2, partial=1, and no=0). The checklist has been ranked in the top six quality assessment tools suitable for use in systematic reviews [1] and has adequate internal consistency, test–retest reliability, inter-rater reliability and criterion validity.

We added the question "Was an attempt made to blind SUD user(s) to the intervention they delivered?", rated either as yes (1) or no/unable to determine (0), to capture performance bias of those implementing SUD reprocessing. We also adapted the scoring for the question "Did the study have sufficient power to detect a clinically important effect where the probability value for a difference being due to chance is less than 5%?", rating as yes (2) where the study was powered to detect a difference for at least one-half of the outcomes, including the primary outcome; partially (1) where the study was powered to detect a difference for the primary outcome only; and no/unable to determine (0) where the study was not powered to detect a difference for any outcome, or we could not tell whether power calculations were undertaken. These adaptations resulted in an overall total possible score of 30. We adapted our quality ratings to allow for the score changes as follows: excellent (27–30), good (21–26), fair (16–20), and poor (≤15); these ratings are in line with previously suggested categories [2].

Details of adaptations to the Consensus Health Economic Criteria list (CHEC-list)

The CHEC-list contains 19 questions on different aspects of economic evaluations: for example, study design; time horizon; study perspective; type of costs and effectiveness measures that are included; the way these costs are measured and valued; incremental analysis of costs and outcomes; discounting; sensitivity analyses; authors' conclusions; and generalisability of study results. Each question can be answered 'yes' or 'no'. If the answer is 'yes', this means that the study either adequately performed the item of concern or reported the item in an appropriate way [3].

The economic studies identified in this systematic review were classified as cost studies rather than full economic evaluations, as study authors used a simple cost-calculator approach where they made various assumptions about the inputs to investigate whether these assumptions affected the overall estimates. Since there are currently no quality appraisal tools specifically designed for these types of studies, adaptations to the CHEC-list were necessary in order to facilitate quality appraisal. Adaptations were made in consultation with two health economists (ÁT and PC) and informed by the Jacobs *et al.* review on this topic which adopted a similar approach to quality appraisal [4]. Specifically, we adapted Question 5 of the CHEC-list to read "Is the chosen time horizon/**duration of study observation period** appropriate to include relevant costs and consequences?" in order to reflect that the time horizon in studies included in this review was derived from the observation period. We removed the questions "Were all outcomes measured appropriately?", "Were all outcomes valued appropriately?", and "Are all future costs and outcomes discounted appropriately?" in line with the Jacobs *et al.* review [4] and given the absence of discounting in these studies. This resulted in a total possible score of 16, with quality ratings of high (>75% of items receiving a score of 1), moderate (between >50% and ≤75% of items receiving a score of 1), and low (≤50% of items receiving a score of 1), which is in keeping with previous research [5–7].

Results of quality appraisals for trials and comparative observational and cost studies

Table S 1 Quality assessment ratings for trials and comparative observational studies

	Cardiac catheters				Implantable cardiac devices			
Item	Browne *et al.* (1997) [8]	Leung *et al.* (2019) [9]	Plante *et al.* (1994) [10]	Unverdorben *et al.* (2005) [11]	Enache *et al.* (2019) [12]	Linde *et al.* (1998) [13]	Nava *et al.* (2013) [14]	Şoşdean *et al.* (2015) [15]
1. Aim/objectives stated	1	1	1	0	1	1	1	1
2. Main outcomes stated before results	0	1	1	1	1	1	1	1
3. Observation characteristics clearly described	0	1	1	1	1	1	1	1
4. Interventions clearly described	1	1	1	1	1	1	1	1
5. Distributions of confounders clearly described	1	2	2	2	2	1	2	2
6. Main findings clearly described	0	0	1	1	0	1	1	1
7. Estimates of random variability	1	1	1	1	1	1	1	1
8. Adverse events reported	1	1	1	1	0	0	1	1
9. Patients lost to	1	0	1	1	0	1	0	0

	Cardiac catheters				Implantable cardiac devices			
follow-up described								
10. Exact probability values reported	0	1	0	1	1	1	1	1
11. Subjects representative of the entire population	1	1	1	1	1	1	1	1
12. Subjects representative of population recruited	0	1	1	0	1	1	1	1
13. Treatment representative of what the majority of patients receive	1	1	1	1	1	1	1	1
14. Attempt made to blind subjects	1	1	1	1	0	0	0	0
15. Attempt made to blind single-use device user(s)	0	0	0	0	0	0	0	0
16. Attempt made to blind those measuring outcomes	1	1	1	0	0	1	1	1
17. 'Data dredging' made clear	0	0	0	1	1	1	1	1
18. Analyses	1	1	1	1	0	1	1	1

	Cardiac catheters				Implantable cardiac devices			
adjusted for follow-up								
19. Statistical tests appropriate	0	1	1	1	1	1	1	1
20. Compliance reliable	1	1	1	1	1	1	1	1
21. Outcome measures accurate	1	1	1	1	1	1	1	1
22. Patients recruited from the same population	1	1	1	1	1	1	1	1
23. Subject recruited over the same period	0	0	1	0	1	1	1	1
24. Randomised	0	0	0	1	0	0	0	0
25. Assignment concealed	0	0	0	0	0	0	0	0
26. Adjustment for confounding	0	1	1	1	0	0	1	1
27. Losses to follow-up considered	1	0	1	0	0	1	1	0
28. Power to detect effect	0	0	0	2	0	0	1	0
**Total score out of 30**	**15**	**20**	**23**	**23**	**17**	**21**	**24**	**22**

Attachment to: McGrath N, Waldron C, Farragher A, Walsh C, Polisena J. Safety, cost and environmental impact of reprocessing high risk single-use medical devices: a systematic review and meta-analysis. GMS Hyg Infect Control. 2025;20:Doc25. DOI: 10.3205/dgkh000554

Table S 2 Quality assessment ratings for cost studies

Cost quality assessment	Cardiac catheters/cannulas	
	Tessarolo *et al.* (2009) [16]	Mak *et al.* (1996) [17]
1. Is the study population clearly described?	0	1
2. Are competing alternatives clearly described?	1	1
3. Is a well-defined research question posed in answerable form?	1	1
4. Is the economic study design appropriate to the stated objective?	1	1
5. Is the chosen time horizon/duration of study observation period appropriate to include relevant costs and consequences?	0	1
6. Is the actual perspective chosen appropriate?	0	1
7. Are all important and relevant costs for each alternative identified?	0	1
8. Are all costs measured appropriately in physical units?	1	0
9. Are costs valued appropriately?	1	1
10. Are all important and relevant safety outcomes for each alternative identified?	0	1
11. Is an incremental analysis of costs and outcomes of alternatives performed?	0	1
12. Are all important variables, whose values are uncertain, appropriately subjected to sensitivity analysis?	1	1
13. Do the conclusions follow from the data reported?	1	1
14. Does the study discuss the generalisability of the results to other settings and patient/client groups?	0	0
15. Does the article indicate that there is no potential conflict of interest of study researcher(s) and funder(s)?	1	0
16. Are ethical and distributional issues discussed appropriately?	0	0
**Total score out of 16**	**8**	**12**

Attachment to: McGrath N, Waldron C, Farragher A, Walsh C, Polisena J. Safety, cost and environmental impact of reprocessing high risk single-use medical devices: a systematic review and meta-analysis. GMS Hyg Infect Control. 2025;20:Doc25. DOI: 10.3205/dgkh000554

References (supplemental files)

1 Deeks JJ, Dinnes J, Abdulnabi R, *et al.* Evaluating non-randomised intervention studies. *Health Technol Assess Winch Engl* 2003;**7**:iii–x, 1–173. doi:https://doi.org/10.3310/hta7270

2 Hooper P, Jutai JW, Strong G, *et al.* Age-related macular degeneration and low-vision rehabilitation: a systematic review. *Can J Ophthalmol J Can Ophtalmol* 2008;**43**:180–7. doi:https://doi.org/10.3129/i08-001

3 Evers S, Goossens M, de Vet H, *et al.* Criteria list for assessment of methodological quality of economic evaluations: Consensus on Health Economic Criteria. *Int J Technol Assess Health Care* 2005;**21**:240–5. doi:https://doi.org/

4 Jacobs P, Polisena J, Hailey D, *et al.* Economic analysis of reprocessing single-use medical devices: a systematic literature review. *Infect Control Hosp Epidemiol* 2008;**29**:297–301. doi:https://doi.org/10.1086/529587

5 Hamberg-van Reenen HH, Proper KI, van den Berg M. Worksite mental health interventions: a systematic review of economic evaluations. *Occup Environ Med* 2012;**69**:837–45. doi:https://doi.org/10.1136/oemed-2012-100668

6 van Dongen J, Proper K, van Wier MF, *et al.* Systematic review on the financial return of worksite health promotion programmes aimed at improving nutrition and/or increasing physical activity. *Obes Rev Off J Int Assoc Study Obes* 2011;**12**:1031–49. doi:https://doi.org/10.1111/j.1467-789X.2011.00925.x

7 Uegaki K, de Bruijne MC, Lambeek L, *et al.* Economic evaluations of occupational health interventions from a corporate perspective - a systematic review of methodological quality. *Scand J Work Environ Health* 2010;**36**:273–88. doi:https://doi.org/10.5271/sjweh.3017

8 Browne K, Maldonado R, Telatnik M, *et al.* Initial experience with reuse of coronary angioplasty catheters in the United States. *J Am Coll Cardiol* 1997;**30**:1735–40. doi:https://doi.org/10.1016/S0735-1097(97)00362-8

9 Leung L, Evranos B, Grimster A, *et al.* Remanufactured circular mapping catheters: safety, effectiveness and cost. *J Interv Card Electrophysiol* 2019;**56**:205–11. doi:https://doi.org/10.1007/s10840-018-0497-x

10 Plante S, Strauss BH, Goulet G, *et al.* Reuse of balloon catheters for coronary angioplasty: A potential cost-saving strategy? *J Am Coll Cardiol* 1994;**24**:1475–81. doi:https://doi.org/10.1016/0735-1097(94)90142-2

11 Unverdorben M, Degenhardt R, Erny D, *et al.* Clinical and angiographic procedural and mid-term outcome with new versus reused balloon catheters in percutaneous coronary interventions. *Indian Heart J* 2005;**57**:114–20. doi:https://doi.org/

12 Enache B, Șoșdean R, Macarie R, *et al.* Assessing the safety of implantable cardioverter-defibrillator reuse—A retrospective case-control study. *PACE - Pacing Clin Electrophysiol* Published Online First: 2019. doi:https://doi.org/10.1111/pace.13742

13 Linde C, Bocray A, Jonsson H. Re-used pacemakers: as safe as new? A retrospective case-control study. *Eur Heart J* 1998;**19**:154–7. doi:https://doi.org/10.1053/euhj.1997.0728

14 Nava S, Morales J, Márquez M, *et al.* Reuse of pacemakers: comparison of short and long-term performance. *Circulation* 2013;**127**:1177-1183. doi:https://doi.org/10.1161/CIRCULATIONAHA.113.001584

15 Şoşdean R, Mornoş C, Enache B, *et al.* Safety and feasibility of biventricular devices reuse in general and elderly population – A single-center retrospective cohort study. *Clin Interv Aging* 2015;**10**:1311–8. doi:https://doi.org/10.2147/CIA.S88805

16 Tessarolo F, Disertori M, Guarrera GM, *et al.* Reprocessing single-use cardiac catheters for interventional cardiology. A cost-minimization model for estimating potential saving at departmental scale and national level. *Ital J Public Health* 2009;**6**:140–9.

17 Mak K-H, Eisenberg MJ, Eccleston DS, *et al.* Cost-efficacy modeling of catheter reuse for percutaneous transluminal coronary angioplasty. *J Am Coll Cardiol* 1996;**28**:106–11. doi:https://doi.org/10.1016/0735-1097(96)00097-6
