# Supplementary material for: Safety, cost and environmental impact of reprocessing high risk single-use medical devices: a systematic review and meta-analysis
Source: GMS Hyg Infect Control. 2025 Jun 6;20:Doc25. doi: 10.3205/dgkh000554 (PMC12248001; doi:10.3205/dgkh000554)
Supplement: Meta-analysis feasibility assessment [file HIC-20-25-s-005.pdf]

Attachment 5 Meta-analysis feasibility assessment

Table S 1 Feasibility assessment for meta-analysis of individual outcomes

Outcome	Number of studies (>3)	Low assessment of quality or of risk of bias (bias in blinding, randomisation, missing outcome data, outcome assessment)	Population, intervention, comparator, outcome(s), time frame, and study design (PICOTS) assessment (clinical and methodological diversity)					Meta-analysis feasibility decision
			Population (eligibility, key demographics)	Intervention and comparator	Outcome (definition and means of reporting)	Study design	Time frame	
Implantable cardiac devices								
Infections	4 studies:<br>Enache *et al.* (2019) [111]<br>Nava *et al.* (2013) [113]<br>Linde *et al.* (1998) [112]<br>Şoşdean *et al.* (2015) [114]	Similar<br>Enache *et al.* (2019): 17/30<br>Linde *et al.* (1998): 21/30<br>Nava *et al.* (2013): 24/30<br>Şoşdean *et al.* (2015): 22/30	Similar eligibility<br>Enache *et al.* (2019): All patients for whom the device was indicated<br>Linde *et al.* (1998): As above, and only patients for whom life expectancy was estimated to be lower than that of the pacemaker received a reprocessed device<br>Nava *et al.* (2013): All patients aged 18 years and over with an indication for pacing<br>Şoşdean *et al.* (2015): Patients requiring implantation with biventricular devices	Similar devices/procedures<br>Enache *et al.* (2019): Implantable cardioverter defibrillators<br>Nava *et al.* (2013): Pacemaker<br>Linde *et al.* (1998): Pacemaker<br>Şoşdean *et al.* (2015): Biventricular devices (pacemakers or defibrillators)<br><br>Location similar<br>Enache *et al.* (2019): Unclear, likely internal	Similar definitions (except Şoşdean *et al.* (2015))<br>Enache *et al.* (2019): Infections that required reintervention<br>Linde *et al.* (1998): Infections that required antibiotics and/or reoperations<br>Nava *et al.* (2013): I: Right endocarditis with electrode involvement; II: Sepsis without evidence of involvement of the	Similar designs<br>Enache *et al.* (2019): Retrospective cohort<br>Linde *et al.* (1998): Retrospective case-matched<br>Nava *et al.* (2013): case matched prospective and retrospective<br>Şoşdean *et al.* (2015):	Similar (except Nava *et al.* (2013))<br>Enache *et al.* (2019): 1–108 months (1 month, 3 months, every 6 months), average 33 months<br>Linde *et al.* (1998): 32 months (±11 months)<br>Nava *et al.* (2013): Not reported	Meets criteria for meta-analysis

Attachment to: McGrath N, Waldron C, Farragher A, Walsh C, Polisena J. Safety, cost and environmental impact of reprocessing high risk single-use medical devices: a systematic review and meta-analysis. GMS Hyg Infect Control. 2025;20:Doc25. DOI: 10.3205/dgkh000554

			Different demographics (Gender only – Enache)<br>Age: Enache *et al.* (2019): 52 years<br>Linde *et al.* (1998): 79 years<br>Nava *et al.* (2013): 60 years<br>Şoşdean *et al.* (2015): 62 years<br>% female: Enache *et al.* (2019): 25%<br>Linde *et al.* (1998): 55%<br>Nava *et al.* (2013): 46%<br>Şoşdean *et al.* (2015): 85%	Linde *et al.* (1998): Internal<br>Nava *et al.* (2013): Internal<br>Şoşdean *et al.* (2015): Likely internal<br><br>Same number of reprocessing cycles<br>Enache *et al.* (2019): 1<br>Linde *et al.* (1998): 1<br>Nava *et al.* (2013): 1<br>Şoşdean *et al.* (2015): 1	circuit or pocket; III: Infection of the pacemaker pocket; and IV: Extrusion of wires or generator<br>Şoşdean *et al.* (2015): Device-related (not defined)<br><br>Similar reporting<br>Enache *et al.* (2019): n, %, odds ratio (OR), confidence interval (CI)<br>Linde *et al.* (1998): n, %<br>Nava *et al.* (2013): n, %, risk ratio (RR) (adjusted), CI<br>Şoşdean *et al.* (2015): n, OR (adjusted), CI	Retrospective case-matched	Şoşdean *et al.* (2015): Up to 94 months, median 35 months	
Unexpected battery depletion	4 studies:<br>Enache *et al.* (2019) [111]<br>Nava *et al.* (2013) [113]<br>Linde *et al.* (1998) [112]	Similar<br>Enache *et al.* (2019): 17/30<br>Linde *et al.* (1998): 21/30<br>Nava *et al.* (2013): 24/30	Similar eligibility<br>Enache *et al.* (2019): All patients for whom the device was indicated<br>Linde *et al.* (1998): As above, and only patients for whom life expectancy	Similar devices/procedures<br>Enache *et al.* (2019): Implantable cardioverter defibrillators	Broadly similar definitions<br>Enache *et al.* (2019): Replacement due to untimely or	Similar designs<br>Enache *et al.* (2019): Retrospective cohort	Similar<br>Enache *et al.* (2019): 1–108 months (1 month, 3 months, every 6	Meets criteria for meta-analysis

Şoşdean *et al.* (2015) [114]	Şoşdean *et al.* (2015): 22/30	was estimated to be lower than that of the pacemaker received a reprocessed device<br>Nava *et al.* (2013): All patients aged 18 years and over with an indication for pacing<br>Şoşdean *et al.* (2015): Patients requiring implantation with biventricular devices<br><br>Different demographics<br>Age: Enache *et al.* (2019): 52 years<br>Linde *et al.* (1998): 79 years<br>Nava *et al.* (2013): 60 years<br>Şoşdean *et al.* (2015): 62 years<br>% female: Enache *et al.* (2019): 25%<br>Linde *et al.* (1998): 55%<br>Nava *et al.* (2013): 46%<br>Şoşdean *et al.* (2015): 85%	Nava *et al.* (2013): Pacemaker<br>Linde *et al.* (1998): Pacemaker<br>Şoşdean *et al.* (2015): Biventricular devices (pacemakers or defibrillators)<br><br>Location<br>Enache *et al.* (2019): Unclear, likely internal<br>Linde *et al.* (1998): Internal<br>Nava *et al.* (2013): Internal<br>Şoşdean *et al.* (2015): Likely internal<br><br>Same number of reprocessing cycles<br>Enache *et al.* (2019): 1<br>Linde *et al.* (1998): 1<br>Nava *et al.* (2013): 1<br>Şoşdean *et al.* (2015): 1	unexpected battery depletion<br>Linde *et al.* (1998): Replacement due to battery depletion<br>Nava *et al.* (2013): The need to remove or change the device because of unexpected battery depletion. Unexpected battery depletion was defined by study group. For new pacemakers, it was defined as depletion before the 6th year after implantation without relation to high pacing output or abnormal electrode impedances. In reused devices, early battery depletion was defined as	Linde *et al.* (1998): Retrospective case-matched<br>Nava *et al.* (2013): case matched prospective and retrospective<br>Şoşdean *et al.* (2015): Retrospective case-matched	months), average 33 months<br>Linde *et al.* (1998): 32 months (±11 months)<br>Nava *et al.* (2013): Not reported<br>Şoşdean *et al.* (2015): Up to 94 months, median 35 months

					occurring before the 4th year.<br>Şoşdean *et al.* (2015): Early battery depletion – considered as after less than 2 years (24 months)<br><br>Similar reporting<br>Enache *et al.* (2019): N, OR, 95% CI<br>Linde *et al.* (1998): n<br>Nava *et al.* (2013): n, %, RR, 95% CI<br>Şoşdean *et al.* (2015): n, IQR			
Other device malfunctio n	2 studies:<br>Nava *et al.* (2013) [113]<br>Linde *et al.* (1998) [112]	Similar<br>Linde *et al.* (1998): 21/30<br>Nava *et al.* (2013): 24/30	Similar eligibility<br>Linde *et al.* (1998): All patients for whom the device was indicated, and only patients for whom life expectancy was estimated to be lower than that of the pacemaker received a reprocessed device<br>Nava *et al.* (2013): All patients aged 18 years and	Similar devices/procedures<br>Linde *et al.* (1998): Pacemaker<br>Nava *et al.* (2013): Pacemaker<br><br>Same location<br>Linde *et al.* (1998): Internal<br>Nava *et al.* (2013): Internal	Similar definition<br>Nava *et al.* (2013): Suspicion of pacemaker malfunction described in the file or causing replacement<br>Linde *et al.* (1998): Suspicion of pacemaker malfunction	Similar designs<br>Linde *et al.* (1998): Retrospective case-matched<br>Nava *et al.* (2013): NRCT (prospective and retrospective, matched)	Unclear similarity<br>Nava *et al.* (2013): Not reported	Does not meet criteria – too few studies

			over with an indication for pacing<br><br>Different demographics<br>Age: Linde *et al.* (1998): 79 years<br>Nava *et al.* (2013): 60 years<br>% female:<br>Linde *et al.* (1998): 55%<br>Nava *et al.* (2013): 46%	Same number of reprocessing cycles<br>Linde *et al.* (1998): 1<br>Nava *et al.* (2013): 1	described in the file or causing replacement<br><br>Similar reporting<br>Nava *et al.* (2013): n, % (unadjusted)<br>Linde *et al.* (1998): n, % (unadjusted)			
Cardiac catheter devices								
Minor complications (pyrogen reactions (fever, temperature, white blood cell count), creatine kinase, author-labelled minor complications)	3 studies:<br>Plante *et al*. (1994) [118]<br>Browne *et al*. (1997) [115]<br>Leung *et al*. (2019) [116]	2/3 similar<br>Leung *et al*. (2019): 20/30<br>Browne *et al*. (1997): 15/30<br>Plante *et al*. (1994): 23/30	Similar eligibility<br>Plante *et al*. (1994): All patients undergoing coronary angioplasty<br>Browne *et al*. (1997): All patients undergoing coronary angioplasty<br>Leung *et al*. (2019): All patients undergoing elective atrial fibrillation ablation<br><br>Similar demographics<br>Age: Browne *et al*. (1997): 64 years<br>Plante *et al*. (1994): 60 years	Broadly similar devices/procedures<br>Plante *et al*. (1994): Balloon, no brand/coronary angioplasty<br>Browne *et al*. (1997): Angioplasty balloon catheters<br>Leung *et al*. (2019): Circular mapping catheter/elective AF ablation<br><br>Different locations<br>Plante *et al*. (1994): Internal	Similar definition<br>Plante *et al*. (1994): Temperature (>38 °C buccal or 38.5 °C rectal), creatine kinase levels<br>Browne *et al*. (1997): Temperature and white blood cell count, obtained before and 24 hours after the procedure (screen for pyrogen reactions)	Similar designs<br>Plante *et al*. (1994): Observational<br>Browne *et al*. (1997): NRCT, case-matched<br>Leung *et al*. (2019): NRCT, case-matched	Different follow-up times<br>Plante *et al*. (1994): Admission to discharge<br>Browne *et al*. (1997): Admission to discharge<br>Leung *et al*. (2019): 3 months	Does not meet criteria – too few studies

			Leung *et al*. (2019): 66 years<br>% female: Plante *et al*. (1994): 28%<br>Browne *et al*. (1997): 44%<br>Leung *et al*. (2019): 32%	Browne *et al*. (1997): External<br>Leung *et al*. (2019): External<br><br>Unclear similarity for number of reprocessing cycles<br>Plante *et al*. (1994): 1–6 (not reported by cycle)<br>Browne *et al*. (1997): Not reported<br>Leung *et al*. (2019): 1–2	Leung *et al*. (2019): Any pyrexial or infective illness<br><br>Similar reporting<br>Plante *et al*. (1994): n, %<br>Browne *et al*. (1997): n, %<br>Leung *et al*. (2019): n, %			
Major complications (evidence of subsequent myocardial infarction (MI) or requirement for emergent percutaneous or	4 studies:<br>Plante *et al*. (1994) [118]<br>Browne *et al*. (1997) [115]<br>Leung *et al*. (2019) [116]<br>Unverdorben *et al.* [120]	Similar (except Browne *et al*. (1997))<br>Leung *et al*. (2019): 20/30<br>Unverdorben *et al*. (2005): 23/30<br>Browne *et al*. (1997): 15/30<br>Plante *et al*. (1994): 23/30	Similar eligibility<br>Plante *et al*. (1994): All patients undergoing coronary angioplasty<br>Browne *et al*. (1997): All patients undergoing coronary angioplasty<br>Unverdorben *et al*. (2005): Coronary angioplasty patients with coronary artery stenosis of ≥70% and <100%, and a visually estimated maximum lesion length of <20 mm in	Broadly similar devices/procedures<br>Plante *et al*. (1994): Balloon, no brand/coronary angioplasty<br>Unverdorben *et al*. (2005): No brand/coronary angioplasty<br>Browne *et al*. (1997): Angioplasty balloon catheters<br>Leung *et al*. (2019): Circular mapping	Broadly similar definitions<br>Plante *et al*. (1994): Angiographically successful angioplasty of all attempted lesions without in-hospital adverse clinical event (defined as death, MI, stroke, emergency angioplasty, or bypass surgery)	Similar designs<br>Plante *et al*. (1994): Observational<br>Browne *et al*. (1997): NRCT, case-matched<br>Unverdorben *et al*. (2005): RCT<br>Leung *et al*. (2019): NRCT, case-matched	Different follow-up times<br>Plante *et al*. (1994): Admission to discharge<br>Browne *et al*. (1997): Admission to discharge<br>Unverdorben *et al*. (2005): 3 months	Does not meet criteria for meta-analysis – too few studies after removal of Browne *et al*. (1997) (poor-quality study) and double zero

Attachment to: McGrath N, Waldron C, Farragher A, Walsh C, Polisena J. Safety, cost and environmental impact of reprocessing high risk single-use medical devices: a systematic review and meta-analysis. GMS Hyg Infect Control. 2025;20:Doc25. DOI: 10.3205/dgkh000554

surgical revascularisation of the target vessel, death, other complications (thrombus; acute and subacute MI))		association with angina pectoris<br>Leung *et al*. (2019): All patients undergoing elective AF ablation<br><br>Similar demographics<br>Age: Browne *et al*. (1997): 64 years<br>Plante *et al*. (1994): 60 years<br>Unverdorben *et al*. (2005): 66 years<br>Leung *et al*. (2019): 66 years<br>% female: Plante *et al*. (1994): 28%<br>Browne *et al*. (1997): 44%<br>Unverdorben *et al*. (2005): 23%<br>Leung *et al*. (2019): 32%	catheter/elective AF ablation<br><br>Different locations<br>Plante *et al*. (1994): Internal<br>Browne *et al*. (1997): External<br>Unverdorben *et al*. (2005): Internal<br>Leung *et al*. (2019): External<br><br>Unclear similarity for number of reprocessing cycles<br>Plante *et al*. (1994): 1–6 (not reported by cycle)<br>Browne *et al*. (1997): Not reported<br>Unverdorben *et al*. (2005): 1–3<br>Leung *et al*. (2019): 1–2	Browne *et al*. (1997): Evidence of subsequent MI or requirement for emergent percutaneous or surgical revascularisation of the target vessel, and death<br>Unverdorben *et al*. (2005): Q-wave MI was diagnosed with the occurrence of new Q-waves (>0.04 seconds) and rise of creatine kinase twice the upper limit of normal with significant increase in creatine kinase whereas in non-Q-wave MIs, pathological Q-waves were absent<br>Leung *et al*. (2019): Evidence of complications of the procedure		Leung *et al*. (2019): 3 months	event studies

					Similar reporting<br>Plante *et al*. (1994): n, %<br>Browne *et al*. (1997): n, %<br>Unverdorben *et al*. (2005): n, %<br>Leung *et al*. (2019): n, %			
Procedure time	4 studies:<br>Plante *et al*. (1994) [118]<br>Browne *et al*. (1997) [115]<br>Leung *et al*. (2019) [116]<br>Unverdorben *et al.* [120]	Similar (except Browne *et al*. (1997))<br>Leung *et al*. (2019): 20/30<br>Unverdorben *et al*. (2005): 23/30<br>Browne *et al*. (1997): 15/30<br>Plante *et al*. (1994): 23/30	Similar eligibility<br>Plante *et al*. (1994): All patients undergoing coronary angioplasty<br>Browne *et al*. (1997): All patients undergoing coronary angioplasty<br>Unverdorben *et al*. (2005): Coronary angioplasty patients with coronary artery stenosis of ≥70% and <100%, and a visually estimated maximum lesion length of <20 mm in association with angina pectoris<br>Leung *et al*. (2019): All patients undergoing elective AF ablation<br><br>Similar demographics	Broadly similar devices/procedures<br>Plante *et al*. (1994): Balloon, no brand/coronary angioplasty<br>Unverdorben *et al*. (2005): No brand/coronary angioplasty<br>Browne *et al*. (1997): Angioplasty balloon catheters<br>Leung *et al*. (2019): Circular mapping catheter/elective AF ablation<br><br>Different locations<br>Plante *et al*. (1994): Internal	Same definition<br><br>Similar reporting (minutes)<br>Plante *et al*. (1994): µ, SD<br>Browne *et al*. (1997): µ, SD<br>Unverdorben *et al*. (2005): µ, SD<br>Leung *et al*. (2019): µ, SD	Similar designs<br>Plante *et al*. (1994): Observational<br>Browne *et al*. (1997): NRCT, case-matched<br>Unverdorben *et al*. (2005): RCT<br>Leung *et al*. (2019): NRCT, case-matched	Same time frame (procedure duration)	Does not meet criteria for meta-analysis – non-normally distributed data

			Age: Browne *et al*. (1997): 64 years<br>Plante *et al*. (1994): 60 years<br>Unverdorben *et al*. (2005): 66 years<br>Leung *et al*. (2019): 66 years<br>% female: Plante *et al*. (1994): 28%<br>Browne *et al*. (1997): 44%<br>Unverdorben *et al*. (2005): 23%<br>Leung *et al*. (2019): 32%	Browne *et al*. (1997): External<br>Unverdorben *et al*. (2005): Internal<br>Leung *et al*. (2019): External<br><br>Unclear similarity for number of reprocessing cycles<br>Plante *et al*. (1994): 1–6 (not reported by cycle)<br>Browne *et al*. (1997): Not reported<br>Unverdorben *et al*. (2005): 1–3<br>Leung *et al*. (2019): 1–2				
Fluoroscopy time	4 studies: Plante *et al*. (1994) [118]<br>Browne *et al*. (1997) [115]<br>Leung *et al*. (2019) [116]<br>Unverdorben *et al.* [120]	Similar (except Browne *et al*. (1997))<br>Leung *et al*. (2019): 20/30<br>Unverdorben *et al*. (2005): 23/30<br>Browne *et al*. (1997): 15/30<br>Plante *et al*. (1994): 23/30	Similar eligibility<br>Plante *et al*. (1994): All patients undergoing coronary angioplasty<br>Browne *et al*. (1997): All patients undergoing coronary angioplasty<br>Unverdorben *et al*. (2005): Coronary angioplasty patients with coronary artery stenosis of ≥70% and <100%, and a visually	Broadly similar devices/procedures<br>Plante *et al*. (1994): Balloon, no brand/coronary angioplasty<br>Unverdorben *et al*. (2005): No brand/coronary angioplasty	Same definition (fluoroscopy time)<br><br>Same reporting (minutes)<br>Browne *et al*. (1997): µ, SD<br>Plante *et al*. (1994): µ, SD<br>Unverdorben *et al*. (2005): µ, SD	Similar designs<br>Plante *et al*. (1994): Observational<br>Browne *et al*. (1997): NRCT, case-matched<br>Unverdorben *et al*. (2005): RCT	Same time frame (during procedure)	Does not meet criteria for meta-analysis – non-normally distributed data

Attachment to: McGrath N, Waldron C, Farragher A, Walsh C, Polisena J. Safety, cost and environmental impact of reprocessing high risk single-use medical devices: a systematic review and meta-analysis. GMS Hyg Infect Control. 2025;20:Doc25. DOI: 10.3205/dgkh000554

estimated maximum lesion length of <20 mm in association with angina pectoris<br>Leung *et al*. (2019): All patients undergoing elective AF ablation	Browne *et al*. (1997): Angioplasty balloon catheters<br>Leung *et al*. (2019): Circular mapping catheter/elective AF ablation	Leung *et al*. (2019): μ, SD	Leung *et al*. (2019): NRCT, case-matched
Similar demographics<br>Age: Browne *et al*. (1997): 64 years<br>Plante *et al*. (1994): 60 years<br>Unverdorben *et al*. (2005): 66 years<br>Leung *et al*. (2019): 66 years<br>% female: Plante *et al*. (1994): 28%<br>Browne *et al*. (1997): 44%<br>Unverdorben *et al*. (2005): 23%<br>Leung *et al*. (2019): 32%	Different locations<br>Plante *et al*. (1994): Internal<br>Browne *et al*. (1997): External<br>Unverdorben *et al*. (2005): Internal<br>Leung *et al*. (2019): External<br><br>Unclear similarity for number of reprocessing cycles<br>Plante *et al*. (1994): 1–6 (not reported by cycle)<br>Browne *et al*. (1997): Not reported<br>Unverdorben *et al*. (2005): 1–3<br>Leung *et al*. (2019): 1–2		

Contrast used	3 studies: Plante *et al*. (1994) [118] Browne *et al*. (1997) [115] Unverdorben *et al.* [120]	2/3 similar Unverdorben *et al*. (2005): 23/30 Browne *et al*. (1997): 15/30 Plante *et al*. (1994): 23/30	Similar eligibility<br>Plante *et al*. (1994): All patients undergoing coronary angioplasty<br>Browne *et al*. (1997): All patients undergoing coronary angioplasty<br>Unverdorben *et al*. (2005): Coronary angioplasty patients with coronary artery stenosis of ≥70% and <100%, and a visually estimated maximum lesion length of <20 mm in association with angina pectoris<br><br>Similar demographics<br>Age: Browne *et al*. (1997): 64 years<br>Plante *et al*. (1994): 60 years<br>Unverdorben *et al*. (2005): 66 years<br>% female: Plante *et al*. (1994): 28%<br>Browne *et al*. (1997): 44%<br>Unverdorben *et al*. (2005): 23%	Broadly similar devices/procedures<br>Plante *et al*. (1994): Balloon, no brand/coronary angioplasty<br>Unverdorben *et al*. (2005): No brand/coronary angioplasty<br>Browne *et al*. (1997): Angioplasty balloon catheters<br><br>Different locations<br>Plante *et al*. (1994): Internal<br>Browne *et al*. (1997): External<br>Unverdorben *et al*. (2005): Internal<br><br>Unclear similarity for number of reprocessing cycles<br>Plante *et al*. (1994): 1–6 (not reported by cycle)<br>Browne *et al*. (1997): Not reported	Definition<br>Unverdorben *et al*. (2005): Not reported<br>Plante *et al*. (1994): Volume of contrast medium used<br>Browne *et al*. (1997): Dye volume<br><br>Similar reporting (mL)<br>Unverdorben *et al*. (2005): µ, SD<br>Plante *et al*. (1994): µ, SD<br>Browne *et al*. (1997): µ, SD	Similar designs<br>Plante *et al*. (1994): Observational<br>Browne *et al*. (1997): NRCT, case-matched<br>Unverdorben *et al*. (2005): RCT	Same time frame (during procedure)	Does not meet criteria – too few studies, as Browne *et al*. (1997) is excluded due to poor study quality

Unverdorben *et al.*
(2005): 1–3

References (supplemental files)

1 Deeks JJ, Dinnes J, Abdulnabi R, *et al.* Evaluating non-randomised intervention studies. *Health Technol Assess Winch Engl* 2003;**7**:iii–x, 1–173. doi:https://doi.org/10.3310/hta7270

2 Hooper P, Jutai JW, Strong G, *et al.* Age-related macular degeneration and low-vision rehabilitation: a systematic review. *Can J Ophthalmol J Can Ophtalmol* 2008;**43**:180–7. doi:https://doi.org/10.3129/i08-001

3 Evers S, Goossens M, de Vet H, *et al.* Criteria list for assessment of methodological quality of economic evaluations: Consensus on Health Economic Criteria. *Int J Technol Assess Health Care* 2005;**21**:240–5. doi:https://doi.org/

4 Jacobs P, Polisena J, Hailey D, *et al.* Economic analysis of reprocessing single-use medical devices: a systematic literature review. *Infect Control Hosp Epidemiol* 2008;**29**:297–301. doi:https://doi.org/10.1086/529587

5 Hamberg-van Reenen HH, Proper KI, van den Berg M. Worksite mental health interventions: a systematic review of economic evaluations. *Occup Environ Med* 2012;**69**:837–45. doi:https://doi.org/10.1136/oemed-2012-100668

6 van Dongen J, Proper K, van Wier MF, *et al.* Systematic review on the financial return of worksite health promotion programmes aimed at improving nutrition and/or increasing physical activity. *Obes Rev Off J Int Assoc Study Obes* 2011;**12**:1031–49. doi:https://doi.org/10.1111/j.1467-789X.2011.00925.x

7 Uegaki K, de Bruijne MC, Lambeek L, *et al.* Economic evaluations of occupational health interventions from a corporate perspective - a systematic review of methodological quality. *Scand J Work Environ Health* 2010;**36**:273–88. doi:https://doi.org/10.5271/sjweh.3017

8 Browne K, Maldonado R, Telatnik M, *et al.* Initial experience with reuse of coronary angioplasty catheters in the United States. *J Am Coll Cardiol* 1997;**30**:1735–40. doi:https://doi.org/10.1016/S0735-1097(97)00362-8

9 Leung L, Evranos B, Grimster A, *et al.* Remanufactured circular mapping catheters: safety, effectiveness and cost. *J Interv Card Electrophysiol* 2019;**56**:205–11. doi:https://doi.org/10.1007/s10840-018-0497-x

10 Plante S, Strauss BH, Goulet G, *et al.* Reuse of balloon catheters for coronary angioplasty: A potential cost-saving strategy? *J Am Coll Cardiol* 1994;**24**:1475–81. doi:https://doi.org/10.1016/0735-1097(94)90142-2

11 Unverdorben M, Degenhardt R, Erny D, *et al.* Clinical and angiographic procedural and mid-term outcome with new versus reused balloon catheters in percutaneous coronary interventions. *Indian Heart J* 2005;**57**:114–20. doi:https://doi.org/

12 Enache B, Șoșdean R, Macarie R, *et al.* Assessing the safety of implantable cardioverter-defibrillator reuse—A retrospective case-control study. *PACE - Pacing Clin Electrophysiol* Published Online First: 2019. doi:https://doi.org/10.1111/pace.13742

13 Linde C, Bocray A, Jonsson H. Re-used pacemakers: as safe as new? A retrospective case-control study. *Eur Heart J* 1998;**19**:154–7. doi:https://doi.org/10.1053/euhj.1997.0728

14 Nava S, Morales J, Márquez M, *et al.* Reuse of pacemakers: comparison of short and long-term performance. *Circulation* 2013;**127**:1177-1183. doi:https://doi.org/10.1161/CIRCULATIONAHA.113.001584

15 Şoşdean R, Mornoş C, Enache B, *et al.* Safety and feasibility of biventricular devices reuse in general and elderly population – A single-center retrospective cohort study. *Clin Interv Aging* 2015;**10**:1311–8. doi:https://doi.org/10.2147/CIA.S88805

16 Tessarolo F, Disertori M, Guarrera GM, *et al.* Reprocessing single-use cardiac catheters for interventional cardiology. A cost-minimization model for estimating potential saving at departmental scale and national level. *Ital J Public Health* 2009;**6**:140–9.

17 Mak K-H, Eisenberg MJ, Eccleston DS, *et al.* Cost-efficacy modeling of catheter reuse for percutaneous transluminal coronary angioplasty. *J Am Coll Cardiol* 1996;**28**:106–11. doi:https://doi.org/10.1016/0735-1097(96)00097-6
