# Supplementary material for: Safety, cost and environmental impact of reprocessing high risk single-use medical devices: a systematic review and meta-analysis
Source: GMS Hyg Infect Control. 2025 Jun 6;20:Doc25. doi: 10.3205/dgkh000554 (PMC12248001; doi:10.3205/dgkh000554)
Supplement: Grading of Recommendations, Assessment, Development and Evaluations [file HIC-20-25-s-006.pdf]

Attachment 6 - Grading of Recommendations, Assessment, Development and Evaluations

Table S 1 Grading of Recommendations, Assessment, Development and Evaluations (GRADE) table with explanations of ratings for individual domains

Outcome	A priori ranking	Downgrade for					Upgrade for			Final grade
		Risk of bias	Inconsistency	Indirectness	Imprecision	Publication bias	Large consistent effect	Dose response	Confounders only reducing size of effect	
Implantable cardiac devices										
Infections	Low: Observational studies.	Serious limitation – downgrade by one: Result based on studies of serious risk of bias concerns.	No serious limitations – no downgrade: Similar point estimates and overlapping (relatively narrow) confidence intervals. Results of meta-analysis Cochran's Q test (*p* > 0.10) and Higgins's I^2 (<40%) indicated low heterogeneity.	Serious limitation – downgrade by one: Some differences in intervention eligibility (reused devices provided when new devices were unavailable, reused devices provided to patients with low life expectancy, reused devices given to patients who could not afford new devices). Patients were older in the Linde *et al.* study compared with others. The gender breakdown varied	Serious limitation – downgrade by one: Reasonably narrow confidence intervals across all 4 studies (all with appreciable benefit and harm). One of 4 studies (Nava *et al.*) undertook a power calculation (and was adequately powered). Consequently, it was unclear whether other studies were	No serious limitation – no downgrade: Our search is comprehensive. Our findings were unadjusted.	No upgrade: Consistent findings, potential for confounders.	No upgrade: Dose-response not applicable.	No upgrade: No adjustment for confounders.	Very low

Attachment to: McGrath N, Waldron C, Farragher A, Walsh C, Polisena J. Safety, cost and environmental impact of reprocessing high risk single-use medical devices: a systematic review and meta-analysis. GMS Hyg Infect Control. 2025;20:Doc25. DOI: 10.3205/dgkh000554

Outcome	A priori ranking	Downgrade for					Upgrade for			Final grade
		Risk of bias	Inconsistency	Indirectness	Imprecision	Publication bias	Large consistent effect	Dose response	Confounders only reducing size of effect	
				across studies ranging from 25% - 85% female. 3/4 studies did not report device brands.	adequately powered.					
Unexpected battery depletion	Low: Two observational studies.	No serious limitations – no downgrade: Result based on low risk of bias for all studies for this outcome.	No serious limitations – no downgrade: Similar point estimates and overlapping (relatively narrow) confidence intervals.	No serious limitations – no downgrade: Some differences in study population (eligibility, age, gender) and intervention (device brands and reprocessing location) unlikely to seriously influence this outcome.	Very serious limitation – downgrade by two: Wide confidence interval across 2 studies with events. One of 2 studies (Nava *et al.*) undertook a power calculation (and was adequately powered). Consequently, it was unclear whether other studies were adequately powered.	No serious limitations – no downgrade: Our search is comprehensive. Our findings were unadjusted.	No upgrade: Inconsistent findings.	No upgrade: Dose-response not applicable.	No upgrade: No adjustment for confounders.	Very low

Attachment to: McGrath N, Waldron C, Farragher A, Walsh C, Polisena J. Safety, cost and environmental impact of reprocessing high risk single-use medical devices: a systematic review and meta-analysis. GMS Hyg Infect Control. 2025;20:Doc25. DOI: 10.3205/dgkh000554

Outcome	A priori ranking	Downgrade for					Upgrade for			Final grade
		Risk of bias	Inconsistency	Indirectness	Imprecision	Publication bias	Large consistent effect	Dose response	Confounders only reducing size of effect	
Cardiac catheters/cannulas										
Major complications	Low:<br>Three of four studies are observational.	Serious limitation – downgrade by one:<br>Result based on serious concerns with respect to risk of bias in three of four studies in relation to this outcome.	No serious limitations – no downgrade:	Serious limitation – downgrade by one:<br>Some differences in procedures (coronary angioplasty vs elective atrial fibrillation ablation). Three of four studies didn't report device brands. Devices were reprocessed externally in three of four studies.	Very serious limitation – downgrade by two:<br>Wide confidence interval across studies with events. One study (Unverdorben *et al.*) undertook a power calculation (and was underpowered for procedure success). Other studies were likely adequately powered but did not undertake a power calculation.	No serious limitations – no downgrade:<br>Our search is comprehensive. Our findings were unadjusted.	No upgrade:<br>Inconsistent findings.	No upgrade:<br>Dose-response not applicable.	No upgrade:<br>No adjustment for confounders	Very low
Total cost difference (per patient)	Low:<br>Observational study	No serious limitation – no	Serious limitation – downgrade by one:<br>One study.	No serious limitations – no downgrade:	Very serious limitation – downgrade by two:	No serious limitations – no downgrade:	No upgrade:<br>One study.	No upgrade:<br>Dose-response not applicable.	No upgrade:<br>No adjustment	Very low

Attachment to: McGrath N, Waldron C, Farragher A, Walsh C, Polisena J. Safety, cost and environmental impact of reprocessing high risk single-use medical devices: a systematic review and meta-analysis. GMS Hyg Infect Control. 2025;20:Doc25. DOI: 10.3205/dgkh000554

Outcome	A priori ranking	Downgrade for					Upgrade for			Final grade
		Risk of bias	Inconsistency	Indirectness	Imprecision	Publication bias	Large consistent effect	Dose response	Confounders only reducing size of effect	
		downgrade: Moderate risk of bias in two domains.		Comparable population for intervention and comparison	Not reported.	Our search is comprehensive. Our findings were unadjusted.			for confounders	

References (supplemental files)

1 Deeks JJ, Dinnes J, Abdulnabi R, *et al.* Evaluating non-randomised intervention studies. *Health Technol Assess Winch Engl* 2003;**7**:iii–x, 1–173. doi:https://doi.org/10.3310/hta7270

2 Hooper P, Jutai JW, Strong G, *et al.* Age-related macular degeneration and low-vision rehabilitation: a systematic review. *Can J Ophthalmol J Can Ophtalmol* 2008;**43**:180–7. doi:https://doi.org/10.3129/i08-001

3 Evers S, Goossens M, de Vet H, *et al.* Criteria list for assessment of methodological quality of economic evaluations: Consensus on Health Economic Criteria. *Int J Technol Assess Health Care* 2005;**21**:240–5. doi:https://doi.org/

4 Jacobs P, Polisena J, Hailey D, *et al.* Economic analysis of reprocessing single-use medical devices: a systematic literature review. *Infect Control Hosp Epidemiol* 2008;**29**:297–301. doi:https://doi.org/10.1086/529587

5 Hamberg-van Reenen HH, Proper KI, van den Berg M. Worksite mental health interventions: a systematic review of economic evaluations. *Occup Environ Med* 2012;**69**:837–45. doi:https://doi.org/10.1136/oemed-2012-100668

6 van Dongen J, Proper K, van Wier MF, *et al.* Systematic review on the financial return of worksite health promotion programmes aimed at improving nutrition and/or increasing physical activity. *Obes Rev Off J Int Assoc Study Obes* 2011;**12**:1031–49. doi:https://doi.org/10.1111/j.1467-789X.2011.00925.x

7 Uegaki K, de Bruijne MC, Lambeek L, *et al.* Economic evaluations of occupational health interventions from a corporate perspective - a systematic review of methodological quality. *Scand J Work Environ Health* 2010;**36**:273–88. doi:https://doi.org/10.5271/sjweh.3017

8 Browne K, Maldonado R, Telatnik M, *et al.* Initial experience with reuse of coronary angioplasty catheters in the United States. *J Am Coll Cardiol* 1997;**30**:1735–40. doi:https://doi.org/10.1016/S0735-1097(97)00362-8

9 Leung L, Evranos B, Grimster A, *et al.* Remanufactured circular mapping catheters: safety, effectiveness and cost. *J Interv Card Electrophysiol* 2019;**56**:205–11. doi:https://doi.org/10.1007/s10840-018-0497-x

10 Plante S, Strauss BH, Goulet G, *et al.* Reuse of balloon catheters for coronary angioplasty: A potential cost-saving strategy? *J Am Coll Cardiol* 1994;**24**:1475–81. doi:https://doi.org/10.1016/0735-1097(94)90142-2

11 Unverdorben M, Degenhardt R, Erny D, *et al.* Clinical and angiographic procedural and mid-term outcome with new versus reused balloon catheters in percutaneous coronary interventions. *Indian Heart J* 2005;**57**:114–20. doi:https://doi.org/

12 Enache B, Șoșdean R, Macarie R, *et al.* Assessing the safety of implantable cardioverter-defibrillator reuse—A retrospective case-control study. *PACE - Pacing Clin Electrophysiol* Published Online First: 2019. doi:https://doi.org/10.1111/pace.13742

13 Linde C, Bocray A, Jonsson H. Re-used pacemakers: as safe as new? A retrospective case-control study. *Eur Heart J* 1998;**19**:154–7. doi:https://doi.org/10.1053/euhj.1997.0728

14 Nava S, Morales J, Márquez M, *et al.* Reuse of pacemakers: comparison of short and long-term performance. *Circulation* 2013;**127**:1177-1183. doi:https://doi.org/10.1161/CIRCULATIONAHA.113.001584

15 Şoşdean R, Mornoş C, Enache B, *et al.* Safety and feasibility of biventricular devices reuse in general and elderly population – A single-center retrospective cohort study. *Clin Interv Aging* 2015;**10**:1311–8. doi:https://doi.org/10.2147/CIA.S88805

16 Tessarolo F, Disertori M, Guarrera GM, *et al.* Reprocessing single-use cardiac catheters for interventional cardiology. A cost-minimization model for estimating potential saving at departmental scale and national level. *Ital J Public Health* 2009;**6**:140–9.

17 Mak K-H, Eisenberg MJ, Eccleston DS, *et al.* Cost-efficacy modeling of catheter reuse for percutaneous transluminal coronary angioplasty. *J Am Coll Cardiol* 1996;**28**:106–11. doi:https://doi.org/10.1016/0735-1097(96)00097-6
